# Homogeneous Electrocatalytic Reduction of Nitrate by an Iron Complex in Water

**DOI:** 10.1021/jacsau.5c00361

**Published:** 2025-07-14

**Authors:** Kaye L. Kuphal, Jesse R. Stroka, María Fernanda Lizarazo, Shamitri Bandyopadhyay, William W. Brennessel, Kara L. Bren

**Affiliations:** Department of Chemistry, 6927 University of Rochester, Rochester, New York 14627-0216, United States

**Keywords:** macrocyclic pyridine(diimine), Faradaic efficiency, 3-(*N*-morpholino)propanesulfonic acid (MOPS)

## Abstract

An iron complex of the macrocyclic pyridine­(diimine) ligand 2,13-dimethyl-3,6,9,12,18-pentaazabicyclo[12.3.1]­octadeca-1(18),2,12,14,16-pentaene (**FeN**_**5**_ **H**_**2**_) is demonstrated as a homogeneous electrocatalyst in a system with a mercury working electrode for multielectron nitrate reduction in buffered water near neutral pH. At −1.4 V vs Ag/AgCl (1 M KCl), the primary products detected are hydroxylamine (15% Faradaic efficiency) and ammonium (40% Faradaic efficiency), along with small quantities of nitrous oxide. MOPS (3-(*N*-morpholino)­propanesulfonic acid) buffer is shown to enhance activity unless present at high concentrations.

## Graphical abstract

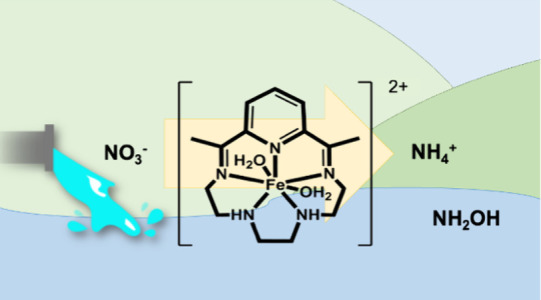

## Introduction

Nitrogen is an essential element in organisms, for which its bioavailability can limit growth.^1^ The use of fertilizers has doubled the input of fixed nitrogen into the biosphere, disrupting the nitrogen cycle (Scheme 1), leading to eutrophication, algal blooms, and hypoxic dead zones, of which there are over 500 worldwide.^2^ Much of the reactive nitrogen in the environment is introduced as ammonium and nitrate.^3^ Ammonium is oxidized to nitrate through microbial nitrification (one possible pathway shown by the gray arrow in Scheme 1).^4–6^ Nitrate is converted to dinitrogen along the denitrification pathway (blue arrows) or to ammonium, via nitrite, through the ammonification pathway (orange arrow).^5^ Although reduction of nitrate is thermodynamically favorable, nitrate accumulates in the environment due to its kinetic stability.^4^ Along with its water solubility, this leads to its accumulation in groundwater, positioning it as a major water pollutant.^7^ These problems have stimulated research in nitrate activation and catalytic nitrate reduction.^6,8^

**Scheme 1:**
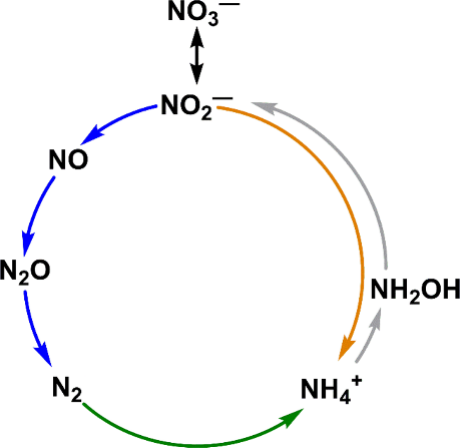
Selected Pathways of the Nitrogen Cycle: Denitrification (blue arrows), Nitrite Reduction to Ammonium (Orange Arrow), Nitrification (Gray Arrows), Nitrogen Fixation (Green Arrow). Not All Intermediates Are Shown

The development of water-compatible nitrate-reducing catalysts containing abundant, inexpensive metals of low toxicity is of high interest. Most research in this area is focused on solid-state electrocatalysts, which show promising activity, but are difficult to characterize in detail.^9^ Gaining molecular-level insight into mechanisms of nitrate reduction benefits from studies of molecular catalysts with well-defined structures.^8,10^ The activation of nitrate by coordination complexes is made difficult by its delocalized electronic structure and weak coordination properties, especially in aqueous solution. Furthermore, achieving product selectivity is a major challenge.^8,9^ Complexes have been reported that overcome some of these challenges and carry out the stoichiometric^11–15^ and catalytic^16–22^ reduction of nitrate, with cobalt-containing complexes being the most studied.^16,17,20,22^

The development and study of nitrate-reducing complexes active in water is crucial due to nitrate’s high solubility and accumulation in water sources. This study, however, poses challenges. Developing activity near neutral pH is critical to avoid decomposition pathways involving unstable nitrite and hydroxylamine intermediates,^23,24^ but buffer has been reported to inhibit nitrate reduction activity in some cases.^20^ On the other hand, an electrode-adsorbed chromium-containing cyclam was found to be active toward nitrate reduction in the presence of a buffer at pH 4.6.^21^

We previously reported that an iron complex of the macrocyclic pyridine­(diimine) ligand 2,13-dimethyl-3,6,9,12,18-pentaazabicyclo[12.3.1]­octadeca-1(18),2,12,14,16-pentaene (**FeN**_**5**_ **H**_**2**_) (Figure 1) catalyzes the electrochemical reduction of nitrite in neutral buffered water to produce hydroxylamine as a major product.^25^ Subsequent pathways including Fe-catalyzed disproportionation and direct reduction of hydroxylamine at a mercury electrode were found to produce ammonium, dinitrogen, and nitrous oxide.^25^ Another report on this complex revealed two different coordination modes of nitrite (nitro, nitrito) to **FeN**_**5**_ **H**_**2**_ in acetonitrile,^26^ inspiring us to investigate the activation of nitrate, which would coordinate through oxygen. Herein, we report an electrochemical system consisting of **FeN**_**5**_ **H**_**2**_ as a homogeneous catalyst with a mercury working electrode active for the multielectron reduction of nitrate in buffered water near neutral pH.

**Figure 1:**
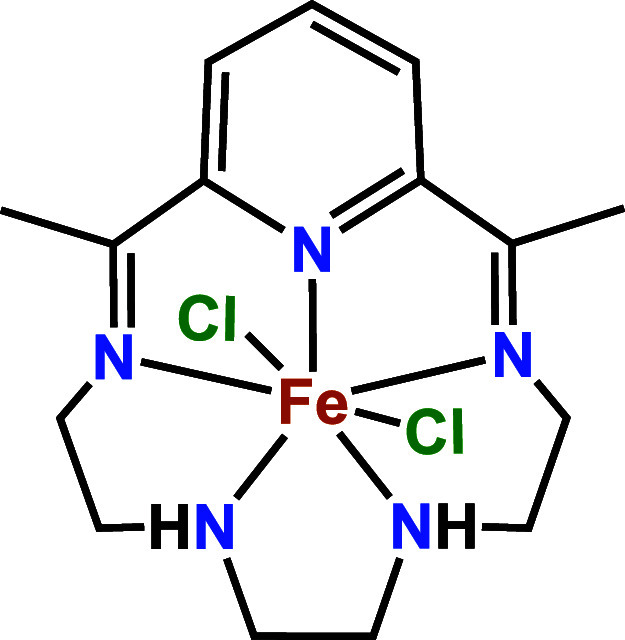
**FeN**_**5**_ **H**_**2**_ as synthesized.

## Results and Discussion

### FeN_5_H_2_ Characterization and Initial Assessment of Catalytic Activity

**FeN**_**5**_ **H**_**2**_ was synthesized as previously described^25^ and characterized by elemental analysis, FTIR spectroscopy (Figure S1), UV–vis absorption spectroscopy (Figure S2), and single-crystal X-ray crystallography (Figures S3–S5). Details of complex preparation and characterization are in the Supporting Information. A hanging mercury drop electrode (HMDE; BASi) was chosen due to its wide cathodic window in water. The redox activity of **FeN**_**5**_ **H**_**2**_ (500 μM) was initially investigated using cyclic voltammetry (CV) in the presence of 100 mM KCl electrolyte and 50 mM 3-(*N*-morpholino)­propanesulfonic acid (MOPS) buffer at pH 7.2. While scanning over the potential range of 0 to −1.35 V vs Ag/AgCl/KCl_(1M)_ (all potentials reported herein are against this reference), two features are observed (Figure 2a). The first is reversible and attributed to the Fe^III/II^ couple of **FeN**_**5**_ **H**_**2**_ at a midpoint potential (*E*_1/2_) of −0.10 V. The second feature is an irreversible wave observed at a half-wave potential (*E*_h_) of −1.20 V and is proposed to result from a small amount of H_2_ production (*vide infra*). Indeed, running controlled potential electrolysis (CPE) of 500 μM FeN_5_H_2_ at −1.5 V in 1 M MOPS yields 20 μmol of H_2_, indicating that H_2_ is a possible product in these reactions (experimental details are in the Supporting Information). We next investigated the electrochemistry of **FeN**_**5**_ **H**_**2**_ in the presence of nitrate as a potential substrate.

**Figure 2:**
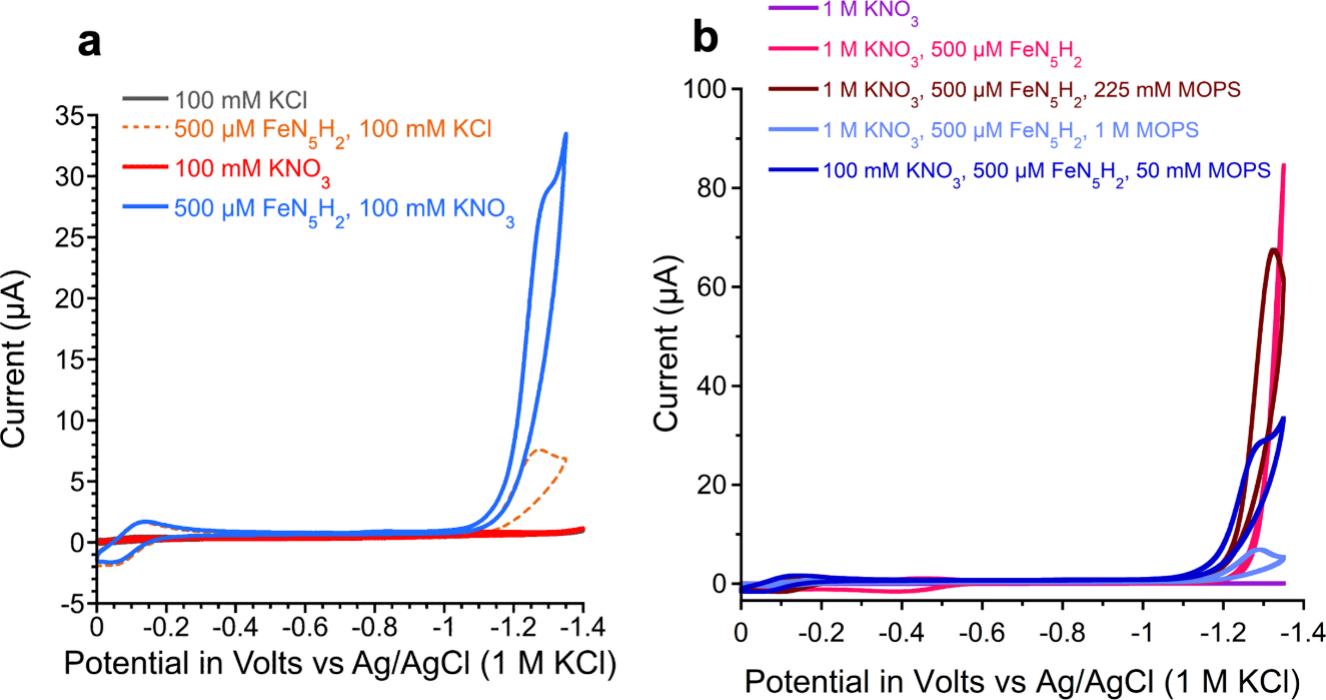
Cyclic voltammograms (100 mV/s) of (a) solutions of 50 mM MOPS at pH 7.2 with KCl, KNO_3_, and/or **FeN**_**5**_ **H**_**2**_ present as indicated in the legend and (b) of solutions at pH 7.2 with MOPS, KNO_3_, and/or **FeN**_**5**_ **H**_**2**_ as indicated in the legend. Scans ran from 0 to negative potential and back to 0 V.

The CV of a solution of 500 μM **FeN**_**5**_ **H**_**2**_ with 100 mM KNO_3_ and 50 mM MOPS at pH 7.2 displays a significant enhancement in current at low potential relative to the CV of **FeN**_**5**_ **H**_**2**_ with KCl rather than KNO_3_, yielding a feature at *E*_h_ = −1.23 V (Figure 2a). The Fe^III/II^ couple at *E*_1/2_ = −0.10 V is unaffected by the presence of nitrate. Furthermore, the UV–vis spectra of **Fe­(III)­N**_**5**_ **H**_**2**_ and **Fe­(II)­N**_**5**_ **H**_**2**_ are unaffected by the presence of nitrate (Figure S6). These observations are consistent with **FeN**_**5**_ **H**_**2**_ having catalytic activity toward nitrate only upon reduction beyond Fe­(II). The irreversible CV feature is sensitive to both KNO_3_ and **FeN**_**5**_ **H**_**2**_ concentration. The peak current increases linearly with [KNO_3_] in the range of 0–600 mM (Figure S7) and with [**FeN**_**5**_ **H**_**2**_] in the range of 0–1 mM (Figure S8).

It is notable that the apparent activity of **FeN**_**5**_ **H**_**2**_ toward nitrate is enhanced in the presence of a buffer (Figure 2b), with peak current increasing from 24 to 58 μA when adding 10 mM MOPS (Figure S9). A complex relationship between peak current and MOPS concentration is observed, which may result from competing effects of MOPS, enhancing proton delivery to the catalyst and potentially inhibiting catalysis at higher concentrations (Figure S9). Indeed, we find that activity is suppressed at high (1 M) MOPS concentrations (Figure 2b, Figure S10). Our previous finding that nitrite can bind to the iron in this complex through an oxygen indeed suggests that iron coordination by a weakly basic group on MOPS to inhibit nitrate activation is possible.^26^ No redox events are observed in the absence of **FeN**_**5**_ **H**_**2**_ (Figure 2b), confirming the role of the complex in the observed activity.

The reactivity of simple iron salts under the same conditions was also investigated in the presence of nitrate to determine whether the catalysis observed is carried out by the **FeN**_**5**_ **H**_**2**_ complex or dissociated iron. From the CV of 1.0 M KNO_3_, 1.0 M MOPS, and 500 μM FeCl_2_ at pH 7.2, no current enhancement at cathodic potentials is observed relative to a CV with no FeCl_2_ (Figure S11). This observation supports the proposal that the observed activity arises from a molecular iron complex.

### Evaluation of Reaction Products

To confirm that electrocatalytic nitrate reduction is taking place and determine the reaction product(s), controlled potential electrolysis (CPE) experiments using a mercury pool electrode were carried out. Initially, a ladder plot was made from 1 min CPE experiments on **FeN**_**5**_ **H**_**2**_ in 1.0 M KNO_3_ or KCl and 1.0 M MOPS at pH 7.2, as well as on 1.0 M KNO_3_ without the iron complex (Figure S10). From these data, we determined that potentials between −1.4 and −1.5 V yielded significant differences in charge passed between samples with and without **FeN**_**5**_ **H**_**2**_, suggesting catalytic activity above background mediated by **FeN**_**5**_ **H**_**2**_ under these conditions. Consequently, these potentials were selected for initial studies. Note that ladder plots collected with lower MOPS concentrations yielded better separation between charge passed with and without the catalyst, but data were collected at higher buffer concentrations to better control pH (Figure S10).

CPE performed at −1.4 V of 1.0 M KNO_3_, 1.0 M MOPS, and 500 μM **FeN**_**5**_ **H**_**2**_ at pH 7.2 passes 83 ± 1 C of charge in 24 h (Figure 3; Figure S12). The reaction solution and headspace were assayed for the possible products ammonium, hydroxylamine, nitric oxide, dinitrogen, nitrous oxide, and nitrite. Ammonium was quantified using the indophenol colorimetric test and confirmed by ^14^N NMR (Figures S13 and S14).^27^ Gas chromatography using a system with a thermal conductivity detector (GC-TCD) was utilized to test the headspace for dinitrogen and nitrous oxide (Figures S15–S17). Hydroxylamine was quantified by titration with [Fe­(CN)_6_]^3–^.^28^ Nitrite was detected and quantified by the Griess colorimetric test (Figure S18), and formation of nitric oxide was investigated using the myoglobin test (Figure S19).^29^ A detailed description of the analyses is included in the Supporting Information, and limits of detection are reported in Table S1. The major products of the reaction, detailed in Table S2, were ammonium at 40 ± 1% Faradaic efficiency (FE) and turnover number (TON) = 17 ± 1 and hydroxylamine at 15 ± 3% FE and TON = 9 ± 2 (errors are determined from duplicate experiments). Small amounts of nitrous oxide and nitrite were detected. Neither nitric oxide nor dinitrogen was detected at levels above background under these conditions. A CPE experiment performed under the same conditions but without **FeN**_**5**_ **H**_**2**_ yielded a much lower charge passed of 10 ± 3 C, and the only product detected was nitrite at 2.2 ± 0.2% FE (Figure 3, Table S2). The activity of **FeN**_**5**_ **H**_**2**_ as a catalyst in this system is clearly evident through the production of significant FEs of hydroxylamine and ammonium in the presence but not in the absence of **FeN**_**5**_ **H**_**2**_ after CPE at −1.4 V.

**Figure 3:**
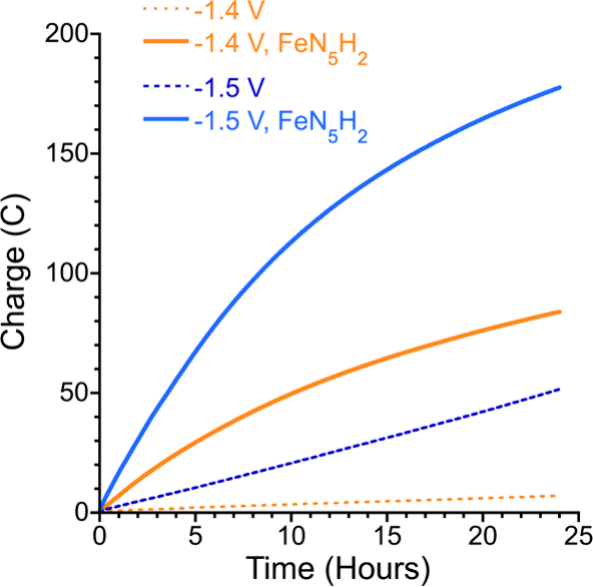
Controlled potential electrolysis of an aqueous solution containing 1.0 M KNO_3_ and 1.0 M MOPS at pH 7.2 for 24 h under a helium headspace. Samples were or without 500 μM **FeN**_**5**_ **H**_**2**_ and applied potential of −1.4 or −1.5 V as indicated in the legend.

When CPE is conducted at −1.5 V on 500 μM **FeN**_**5**_ **H**_**2**_ in 1.0 M KNO_3_ and 1.0 M MOPS at pH 7.2, a considerably larger amount of charge is passed (185 ± 8 C; Figure 3). With the exception of hydroxylamine, which is produced in larger proportions, a similar product distribution is obtained in comparison to the results at −1.4 V, with ammonium at 33 ± 4% FE and TON = 27 ± 6 and hydroxylamine at 26 ± 1% FE and TON = 17 ± 1, as well as small amounts of nitrous oxide and nitrite. At this more cathodic potential, some hydroxylamine and ammonium are produced in the absence of the iron complex. However, activity increases significantly when **FeN**_**5**_ **H**_**2**_ is present. Compared to the results at −1.4 V, all products were obtained in greater molar quantities at −1.5 V (Table S2). It may be surprising that the proportion of ammonium, as the most reduced product, decreases at more cathodic potentials. We propose that this is because a significant amount of the ammonium produced results from electroneutral disproportionation of hydroxylamine catalyzed by **FeN**_**5**_ **H**_**2**_ as previously reported.^25^

The charge passed in the absence of **FeN**_**5**_ **H**_**2**_ was significantly lower compared to when **FeN**_**5**_ **H**_**2**_ is present under both potentials: 10 ± 3 C at −1.4 V and 49.2 ± 2 C at −1.5 V. At −1.4 V, the only detected product was nitrite, at an FE of only 2.2 ± 0.2%, suggesting that the mercury electrode has activity for the reduction of nitrate to nitrite, but reducing nitrate beyond 2 electrons under these conditions requires **FeN**_**5**_ **H**_**2**_. At −1.5 V in the absence of **FeN**_**5**_ **H**_**2**_, both nitrite (3.3 ± 0.5% FE) and hydroxylamine (14 ± 1% FE) are produced (Table S2). As also reported in Table S2, modest changes in pH are observed throughout the CPE experiments, limiting the acidic and basic decomposition pathways available to nitrite and hydroxylamine intermediates and products.^23,24^ Finally, we also conducted CPE experiments under the same conditions but at −1.3 V to determine whether there is activity at this less cathodic potential. Charge passed is substantially lower (16.9 ± 7.8 C) but remains higher than results without **FeN**_**5**_ **H**_**2**_; see details in Table S3. This experiment yields nitrite as the primary product in small amounts.

In the same potential region where an irreversible CV wave is observed with nitrate and catalyst, there is a smaller irreversible peak in a CV with KCl and catalyst (Figure 2a), which we hypothesized to be from a small amount of hydrogen production. To follow up on this observation, 2-h CPE experiments were performed on solutions containing 1.0 M KCl and 1.0 M MOPS with and without **FeN**_**5**_ **H**_**2**_. Results showed that the catalyst has hydrogen production activity, although at a low level, when in solution with KCl and MOPS (Figure S17). For example, when CPE is conducted for 2 h at −1.5 V of 1.0 M KCl, 1.0 M MOPS, and 500 μM **FeN**_**5**_ **H**_2_ at pH 7.2, a small amount of charge is passed (2.4 ± 0.3 C) with 65 ± 4% FE for production of hydrogen (TON = 3.1 ± 0.3, 7.65 ± 1.9 μmol H_2_). Hydrogen production thus likely contributes to the unaccounted FE in the nitrate reduction experiments.^30^

### Comparison to the Activity of Related Systems

The activity of **FeN**_**5**_ **H**_**2**_ at a mercury electrode for nitrate reduction compares well with those of other molecular nitrate-reducing catalysts. Most molecular catalysts for nitrate reduction in water use cobalt, with none previously reported using iron to our knowledge. The most active may be Co­(cyclam) adsorbed on a mercury pool electrode, which performs especially well for nitrate reduction to hydroxylamine, with a TON of 900 at −1.49 V vs Ag/AgCl/KCl_(1M)_ (all reported potentials are converted to this reference). It also yields a TON of 24 for ammonium and 390 for nitrite.^16^ High activity for producing hydroxylamine was also reported at −0.8 V by an a cobalt protoporphyrin immobilized on a pyrolytic graphite electrode at a more cathodic potential of −1.0 V; this system produces mixtures of hydroxylamine and ammonia, but TON values were not reported.^22^ Another active cobalt macrocycle catalyst, [Co­(DIM)­Br_2_]^+^, at a glassy carbon electrode, reduces nitrate to ammonia with an outstanding 97% FE and a TON of 3.4 at an applied potential of −1.0 V under acidic conditions.^20^ A rinse test is consistent with [Co­(DIM)­Br_2_]^+^ acting as a homogeneous catalyst. Another cobalt complex, [Co­(CR)­NO­(Br)]^+^, where CR is a macrocyclic pyridine­(diimine) ligand, catalyzes nitrate reduction at a glassy carbon working electrode at −1.35 V, yielding 57% FE for ammonium (TON 22.6) and a 3% FE for nitrite after 2 h, with no detectable hydroxylamine.^31^ Finally, a chromium cyclam complex adsorbed at a mercury electrode was reported to catalyze nitrate reduction to nitrite (FE = 24%) and ammonia (FE = 17%) at an applied potential of −0.98 V.^21^

Reports of molecular iron catalysts for nitrate reduction are rare, but there are a few examples. A seminal report that is not an electrocatalyst is an iron azafulvene-amine complex with a TON of 3.5 for production of nitric oxide as a sole product of nitrate reduction in acetonitrile.^19^ For an example of an electrocatalytic system, a glassy carbon electrode modified with an iron phthalocyanine film yields ammonia from nitrate at a 55% FE at −1.5 V in basic conditions (0.1 M KOH). **FeN**_**5**_ **H**_**2**_ is unusual for its function in water near neutral pH to facilitate formation of multielectron reduced products. Importantly, it functions in the presence of a buffer, although buffer has previously been reported to inhibit nitrate reduction by a molecular catalyst.^20^ The presence of a buffer not only maintains pH but may also facilitate proton delivery to the catalyst.^25,32–35^ Indeed, we observe **FeN**_**5**_ **H**_**2**_ activation for nitrate reduction by addition of MOPS buffer. At the same time, high concentrations of buffer (1 M) are inhibitory (Figure 2b, Figure S10).

The products of nitrate reduction by **FeN**_**5**_ **H**_**2**_ are similar to those determined for nitrite reduction by the same complex in previous work.^25^ We thus hypothesized that nitrite is an intermediate in nitrate reduction by this system. To test this hypothesis, we performed 1 min CPE experiments at −1.5 V and analyzed the products and found that nitrite was produced when **FeN**_**5**_ **H**_**2**_ was present with an FE of 2.3 ± 0.4% (Table S4). Electrocatalytic reduction of nitrite by **FeN**_**5**_ **H**_**2**_ produces primarily hydroxylamine, and that activity was enabled by MOPS buffer, with no activity in the absence of a buffer and a linear increase in activity with buffer concentration. Upon production of hydroxylamine, two pathways were found to be active to yield additional products: electroneutral disproportionation of hydroxylamine to produce ammonium, nitrous oxide, and dinitrogen catalyzed by **FeN**_**5**_ **H**_**2**_ and low levels of reduction of hydroxylamine to ammonium by the Hg electrode.^25^ The disproportionation pathway is expected to be active in the present system and contributes to producing nitrous oxide and ammonium. The production of dinitrogen from the current nitrate reduction experiments was not confirmed because the low levels of dinitrogen detected were not above background. Considering the results from the current work and our previous study of nitrite reduction by **FeN**_**5**_ **H**_**2**_, we propose that the reaction pathways shown in Scheme 2 are in effect.

**Scheme 2:**
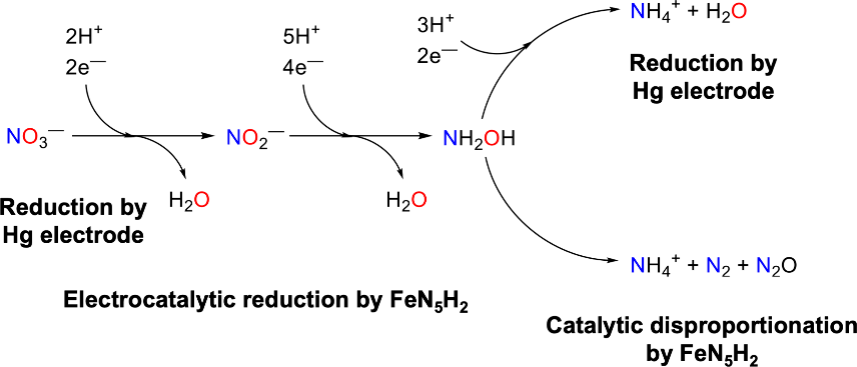
. **FeN**_**5**_ **H**_**2**_ Electrocatalytically Reduces Nitrate to Hydroxylamine with Nitrite as a Proposed Intermediate^a^

a Hydroxylamine can then undergo direct reduction at the Hg electrode to produce ammonium or an electroneutral disproportionation reaction catalyzed by **FeN_5_H_2_** to produce ammonium, nitrous oxide, and likely dinitrogen. Note that some activity for reduction of nitrate to nitrite by the Hg electrode is also seen.

### Evaluation of Catalyst Homogeneity

Catalyst homogeneity was first tested by performing a dip-and-stir test, a rinse test modified for use with a HMDE.^36^ A CV was collected in the presence of 1.0 M KNO_3_, 1.0 M MOPS, and 500 μM **FeN**_**5**_ **H**_**2**_ at pH 7.2 over the potential range of 0 to −1.35 V. Following CV, the mercury drop was retained, and the solution was replaced with one containing 1.0 M KNO_3_ and 1.0 M MOPS at pH 7.2 but no **FeN**_**5**_ **H**_**2**_. CV was repeated over the same potential range, but no current was observed above baseline, indicating that **FeN**_**5**_ **H**_**2**_ did not strongly adsorb to the mercury drop to form a heterogeneous or adsorbed catalyst (Figure S20). Also consistent with homogeneous catalysis is the observation of the expected dependence of CV peak intensity on the scan rate according to the Randles–Sevcik equation for both catalytic and Fe­(III/II) features (Figure S21).^37^

There are concerns that a rinse test can give false negative results, in particular if the catalyst adsorbs weakly or transiently. Furthermore, the scan rate dependence may reflect diffusion of a component of the reaction other than the catalyst itself. Chronoamperometry (CA) allows a more rigorous evaluation of catalyst homogeneity.^37–39^ Adsorption to an electrode is evident in CA by deviations from the expected current response as described by the Cottrell equation (see the Supporting Information).^37^ The key relationship of interest within the Cottrell equation is the current decaying proportionally to the inverse square root of time for a diffusion-limited process.

Here, we performed CA of 500 μM **FeN**_**5**_ **H**_**2**_ in the presence of 1.0 M KNO_3_ and 1.0 M MOPS at pH 7.2 at a mercury pool electrode to assess the electrode adsorption of the metal complex. The potential was stepped into the range of −1.0 to −1.4 V in 25-mV increments to probe the catalytic event observed in CV across a range of potentials. No increase in current indicative of film formation is observed under these conditions (Figure 4). Similarly, CA investigations of the Fe­(III/II) event performed by stepping the potential within the range of 0 to −0.3 V in 25-mV increments support the homogeneity of that process as well (Figure S22). Thus, no evidence of adsorption of **FeN**_**5**_ **H**_**2**_ to the electrode is found for the catalytic or Fe­(III/II) processes.

**Figure 4:**
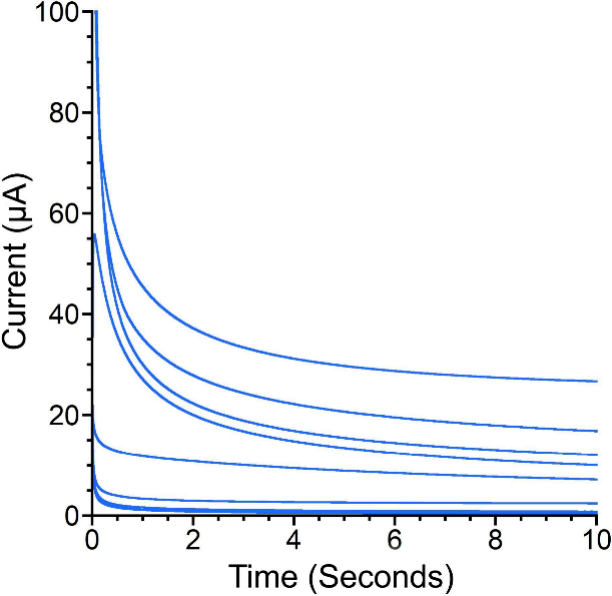
Chronoamperograms of 500 mM **FeN**_**5**_ **H**_**2**_ in 1.0 M KNO_3_ and 1.0 M MOPS at pH 7.2. Potential is stepped from 0 to −1.4 V vs Ag/AgCl/KCl_(1M)_ in 25-mV increments.

To assess the impact of electrolysis at low potentials over time on the integrity of the catalyst, UV–vis spectra of the reaction solution before and after CPE were collected and are shown in Figure S23. The results suggest modification of the catalyst followed by degradation, although even after 24 h the spectra are consistent with persistence of a molecular complex. The blue shift of the visible feature after 2 h is consistent with saturation of the imines.^40^ Attempts to synthesize the saturated version of this Fe complex for comparison were not successful. More detailed mechanistic studies of this system are planned.

## Conclusions

These results demonstrate that **FeN**_**5**_ **H**_**2**_ is a homogeneous molecular electrocatalyst forming a system active for multielectron nitrate reduction to hydroxylamine and ammonia with a mercury electrode. **FeN**_**5**_ **H**_**2**_ has a number of notable features among related catalysts: it contains iron, does not adsorb to the electrode, and operates in the presence of buffer near neutral pH. This last quality allows for relatively constant pH maintenance in water throughout experiments to deter pH-dependent decomposition of intermediates and products. The reactivity is also long-lived, with turnover numbers comparing well to the leading molecular nitrate-reducing catalysts. In addition to the activity of **FeN**_**5**_ **H**_**2**_, reduction of nitrate to nitrite and of hydroxylamine to ammonia by the mercury electrode is expected to contribute to the observed activity of the system. Future efforts toward enhancing system activity will look toward augmenting interactions between the catalyst and the substrate through catalyst design^19^ and facilitating delivery of protons by tuning intramolecular^41^ and intermolecular^25,34^ proton donors.

## Supplementary Material



## Notes

Detailed description of materials and methods, spectroscopic and crystallographic characterization of **FeN**_**5**_ **H**_**2**_, additional electrochemical data, results of product analyses (PDF)
