# Supplementary material for: Homogeneous Electrocatalytic Reduction of Nitrate by an Iron Complex in Water
Source: JACS Au. 2025 Jul 14;5(7):3182–8. doi: 10.1021/jacsau.5c00361 (PMC12308391; doi:10.1021/jacsau.5c00361)
Supplement: Supplementary file 1 [file au5c00361_si_001.pdf]

# Supporting Information

## Homogeneous Electrocatalytic Reduction of Nitrate by an Iron Complex in Water

Kaye L. Kuphal†, Jesse R. Stroka†, María Fernanda Lizarazo, Shamitri Bandyopadhyay, William W. Brennessel, and Kara L. Bren*

Department of Chemistry, University of Rochester, Rochester, NY 14627-0216

†These authors contributed equally

*Corresponding author: kara.bren@rochester.edu

**Table of Contents**

Materials …………………………………………………………………………………… S3

$FeN_5H_2$ Preparation and Characterization ……………………………………………… S3

Single Crystal X-ray Crystallography………………………………………………….... S5

Spectroscopic Methods……………………………………………………………….….. S8

Investigation of Interaction of $FeN_5H_2$ with Nitrate …………………………………... S8

Electrochemical Methods ………………………………………………………………... S9

Product Quantification ……………………………………………………………….... S14

Results of CPE Experiments …………………………………………………………….. S15

Analysis of Catalyst Homogeneity ……………………………………………………….. S22

Crystal Structure Data, Coordinates, and Results ……………………………………….. S24

References ………………………………………………………………………………... S33

## Materials

Doubly deionized water with a resistivity of 18.2 MΩ was used as a solvent in all procedures. All chemicals were used from the manufacturer without additional purification. For the synthesis of **$FeN_5H_2$**, $FeCl_3$ was obtained from Strem Chemicals and 2,6-diactylpyridine and triethylenetetramine were obtained from Sigma-Aldrich. Sodium dithionite to perform the chemical reduction of **$FeN_5H_2$** was obtained from Fisher Chemical. For the reactivity studies, 3-(N-morpholino)propanesulfonate (MOPS) was obtained from Sigma-Aldrich and potassium chloride and potassium nitrate were obtained from Fisher Chemical. For GC-TCD studies, the nitrous oxide and hydrogen standards were of AA grade and obtained from Air Products. For the redox titration, potassium ferricyanide was obtained from Sigma-Aldrich Chemical. For the Griess test, an Invitrogen Griess reagent kit for nitrite quantitation was obtained from ThermoFisher Scientific. For the indophenol test, ammonium sulfate was obtained from Fisher Chemical, phenol from Acros Organics, sodium nitroferricyanide dihydrate from Sigma-Aldrich, and sodium citrate dihydrate from Fisher Chemical. For the myoglobin test to detect nitric oxide, myoglobin was obtained from Sigma-Aldrich and potassium phosphate was obtained from Fisher Chemical. Sodium hydroxide and hydrochloric acid, both from Fisher Chemical, were used for pH adjustments.

## $FeN_5H_2$ Preparation and Characterization

In a round-bottom Schlenk flask, 1.62 g (0.01 mol) of $FeCl_3$ and 1.63 g (0.01 mol) of 1,6-diacetylpyridine are added to 140 mL of 50% ethanol in water. The reaction mixture is stirred for 30 minutes while heating at 70 ºC. Then, 1.54 g (0.01 mol) of triethylenetetramine is added to the reaction mixture followed by 0.8 mL of acetic acid. The reaction is stirred at 70 ºC for 8 hours. Reaction completion is evaluated using Fourier-transform infrared spectroscopy (FTIR) with the disappearance of the carbonyl stretch of 2,6-diacetylpyridine at 1697 $cm^{-1}$ accompanied with the appearance of an imine stretch at 1655 $cm^{-1}$ attributed to the imine bond of **$FeN_5H_2$** (Fig. S1).[1,2] The identity of **$FeN_5H_2$** was confirmed by elemental analysis (CENTC Elemental Analysis Facility, University of Rochester, funded by NSF CHE-0650456), UV-vis absorption spectroscopy (Fig. S2) and crystallography (X-ray Crystallographic Facility, University of Rochester, funded by NSF MRI program grant CHE-1725028) as well as by electronic absorption spectroscopy as

previously detailed.[3,4] Single crystals suitable for X-ray crystallography were obtained by recrystallization by slow diffusion of diethyl ether into acetonitrile. Elemental analysis for $C_{15}$ $H_{27}$ $Cl_2$ Fe $N_5$ $O_6$ (MW = 500.15538 g/mol): Calculated (%): C, 36.021; H, 5.441; N, 14.002. Found (%): C, 35.981; H, 5.093; N, 13.910.

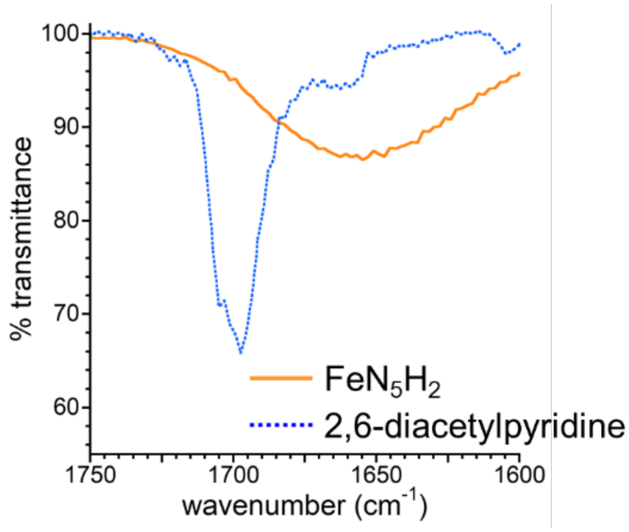


**Figure S1**. FTIR of 2,6-diacetylpyridine displaying its carbonyl stretch at 1697 $cm^{-1}$ (dotted blue trace). A spectrum of the product mixture displaying completion of reaction to form $FeN_5H_2$ with imine stretch at 1655 $cm^{-1}$ shown (solid orange trace).

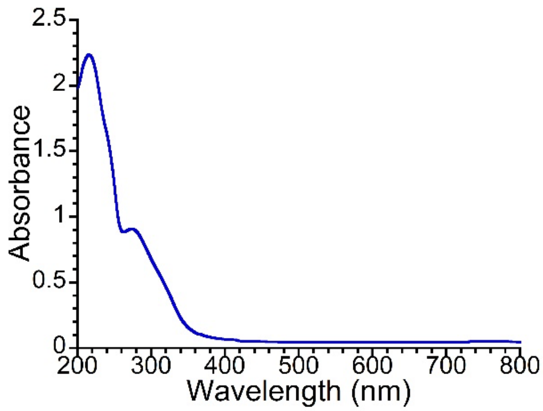


**Figure S2.** UV-vis spectrum of 0.1 mM **Fe(III)$N_5H_2$** in water at pH 7.2.

## Single Crystal X-ray Crystallography

*Data collection*

A crystal (0.154 x 0.128 x 0.056 $mm^3$) was placed onto a thin glass optical fiber or a nylon loop and mounted on a Rigaku XtaLAB Synergy-S Dualflex diffractometer equipped with a HyPix-6000HE HPC area detector for data collection at 100.00(10) K. A preliminary set of cell constants and an orientation matrix were calculated from a small sampling of reflections.[5] A short pre-experiment was run, from which an optimal data collection strategy was determined. The full data collection was carried out using a PhotonJet (Cu) X-ray source with frame times of 1.50 and 5.99 seconds and a detector distance of 34.0 mm. Series of frames were collected in 0.50° steps in *w* at different 2*q*, *k*, and *f* settings. After the intensity data were corrected for absorption, the final cell constants were calculated from the xyz centroids of 18277 strong reflections from the actual data collection after integration.[5] See Tables S4-S10 for detailed crystal and refinement information. The structure was deposited in the Cambridge Crystallographic Data Centre, Deposition number 2435237.

*Structure solution and refinement*

The structure was solved using SHELXT[6] and refined using SHELXL.[7] The space group *Pbca* was determined based on systematic absences. Most or all non-hydrogen atoms were assigned from the solution. Full-matrix least squares / difference Fourier cycles were performed which located any remaining non-hydrogen atoms. All non-hydrogen atoms were refined with anisotropic displacement parameters. All N-H and O-H hydrogen atoms were found from the difference Fourier map and refined freely. All other hydrogen atoms were placed in ideal positions and refined as riding atoms with relative isotropic displacement parameters. The final full matrix least squares refinement converged to $R1 = 0.0376$ ($F^2$, $I > 2s(I)$) and $wR2 = 0.1043$ ($F^2$, all data).

*Structure description*

The structure is the one suggested. The asymmetric unit contains one dicationic iron complex with two axial water ligands, one chloride anion, and one perchlorate anion in general positions (Fig. S3). The perchlorate anion is modeled as disordered over two positions (0.74:0.26). Cations and anions are linked in sheets normal to [001] by N-H and O-H hydrogen bonding (Fig. S4). The

ORTEP diagram is shown in Fig. S5.

Structure manipulation and figure generation were performed using Olex2.[8] Unless noted otherwise all structural diagrams containing anisotropic displacement ellipsoids are drawn at the 50 % probability level.

Data collection, structure solution, and structure refinement were conducted at the X-ray Crystallographic Facility, B04 Hutchison Hall, Department of Chemistry, University of Rochester. The instrument was purchased with funding from NSF MRI program grant CHE-1725028.

*Some equations of interest:*

$R_{int} = S\ |F_o^2 - < F_o^2 >|\ /\ S|F_o^2|$

$R1 = S||F_o|-|F_c||\ /\ S|F_o|$

$wR2 = [S[w(F_o^2-F_c^2)^2]\ /\ S\ [w(F_o^2)^2]]^{1/2}$

where $w = 1\ /\ [s^2\ (F_o^2) + (aP)^2 + bP]$ and

$P = 1/3\ \max\ (0, F_o^2) + 2/3\ F_c^2$

GOF = $S = [S[w(F_o^2-F_c^2)^2]\ /\ (m-n)]^{1/2}$

where $m$ = number of reflections and $n$ = number of parameters

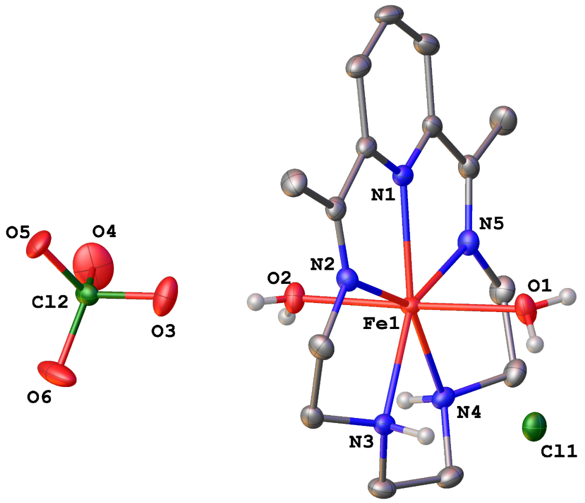


**Figure S3**. Asymmetric unit showing **FeN$_5$H$_2$**, perchlorate, and chloride counterions.

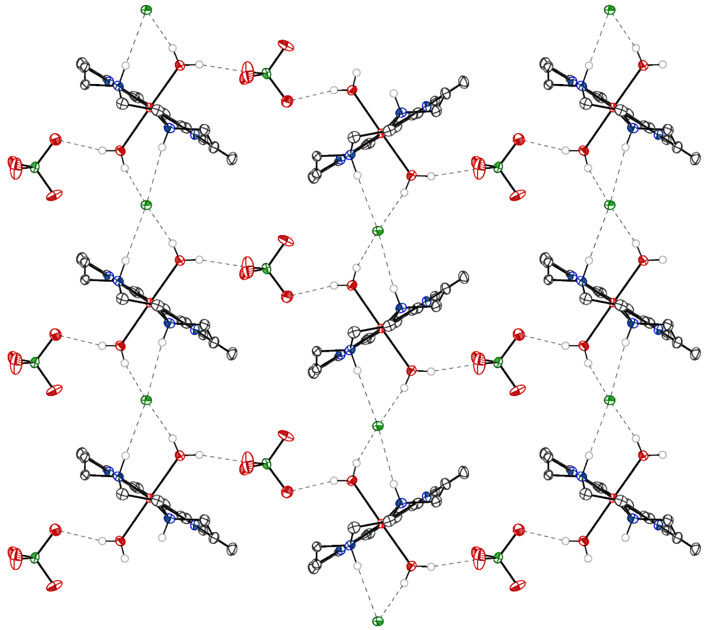

**Figure S4**. Crystal packing of **FeN$_5$H$_2$**, perchlorate, and chloride counterions.

S6).

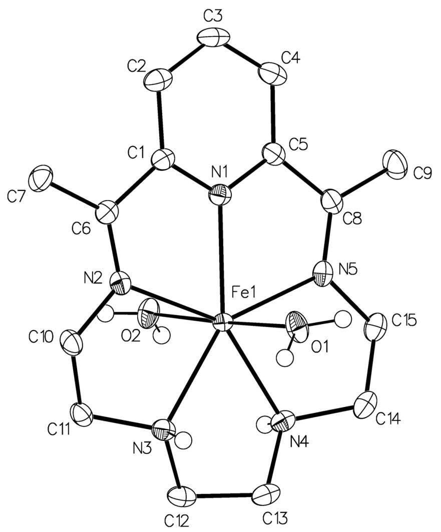


**Figure S5**. ORTEP diagram of **$FeN_5H_2$**

## Spectroscopic Methods

UV-vis spectroscopy was conducted on a Shimadzu UV-2401PC spectrophotometer using a quartz cuvette with a 1.0 cm path length.

FTIR spectroscopy was performed using a Shimadzu IRPrestige-21 spectrophotometer. Measurements were performed using the attenuated total reflectance (ATR) technique with a PIKE Technologies MIRacle Single Reflection Horizontal ATR Accessory attachment.

## Investigation of Interaction of $FeN_5H_2$ with Nitrate

Electronic absorption spectroscopy of $Fe^{III}N_5H_2$ in the presence of nitrate shows no shift in the visible region to indicate the presence of an interaction (Fig. S6). Electrogeneration of $Fe^{II}N_5H_2$ was performed using the spectroelectrochemistry setup described above by applying a potential of –0.3 V vs Ag/AgCl (1.0 M KCl). No changes were observed in the MLCT band of $Fe^{II}N_5H_2$ in the presence of nitrate, which was previously shown to be sensitive to changes in axial ligation (Fig. S6).

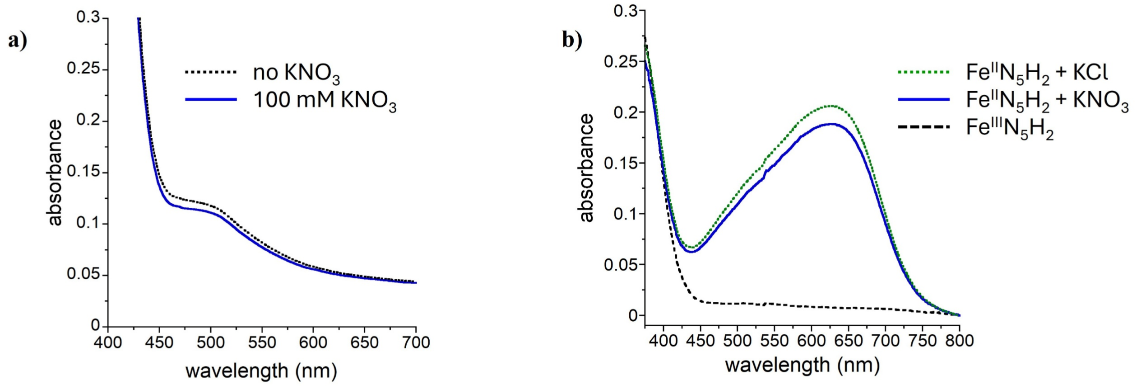


**Figure S6.** a) UV-vis spectra of 5.0 mM $FeN_5H_2$ at pH 7.2 in the absence and presence of 100 mM $KNO_3$ as indicated in the legend. The cuvette path length is 1.0 cm. b) 7.9 mM $FeN_5H_2$ in pH 7.2 water with 100 mM KCl or $KNO_3$ as indicated in the legend. $Fe^{II}N_5H_2$ is electrogenerated by applying a potential of -0.3 V vs Ag/AgCl (1.0 M KCl) at a gold mesh electrode in an optically transparent cell. The cuvette path length is 0.10 cm.

**Electrochemical Methods**

Cyclic voltammetry (CV) experiments were performed on a CH Instruments electrochemical analyzer (CHI620D) potentiostat using ir compensation using the software provided. A three-electrode setup was used, consisting of a 3-mm diameter glassy carbon button counter electrode (CH Instruments), an Ag/AgCl (1.0 M KCl) reference electrode (CH Instruments), and a hanging mercury drop working electrode (HMDE) with a surface area of 0.026 $cm^2$ (BASi). CV was performed on 5.0 mL, $N_2$-sparged aqueous solutions under an $N_2$ atmosphere at 20 ± 2 ºC. The glassy carbon counter electrode was regularly polished between CV experiments using 0.05 μm alumina powder with 100 figure-eights in each direction.

Controlled potential electrolysis (CPE) experiments were performed in custom electrolysis cells (Adams and Chittenden). The cells were composed of two cylindrical chambers of equal volume with 1.5 cm diameter and 6.5 cm height. The chambers are separated by glass frits (P5 porosity). One chamber contains a 1.3 mL mercury pool electrode and an Ag/AgCl (1.0 M KCl) reference electrode (CH instruments). The other chamber contains a glassy carbon rod counter electrode (CH

Instruments). In each experiment, 5.0 mL of solution as added to each chamber. Each chamber was then sealed with a septum and sparged with He gas for 20 minutes to remove oxygen.

Electrogeneration of reduced states of $FeN_5H_2$ was performed using a spectroelectrochemistry setup from BASi consisting of a quartz cuvette with a 0.10 cm path length. A gold mesh working electrode was used (BASi) with an Ag/AgCl (1.0 M KCl) reference electrode (CH Instruments) and a platinum wire counter electrode (BASi).

*Equations of Interest*

Turnover number (TON) was estimated using the following equation:

$TON = n_{product} / n_{FeN5H2}$

where $n_{product}$ = moles of hydroxylamine or ammonium produced and $n_{FeN5H2}$ = moles of $FeN_5H_2$ added.

Turnover frequency (TOF) can then be estimated as follows:

TOF = TON / t

Where TON = turnover number and t = time

Faradaic efficiency (FE) was calculated from the following equation:

$FE (\%) = (n_e \times F \times 100\%) / Q_T$

where $n_e$ = the moles of electrons required to generate the measured moles of a species of interest from $NO_3^-$ (8 moles of electrons per mole of ammonium, or 6 moles of electrons per mole of hydroxylamine), F = Faraday's constant (96485.3 C/mol $e^-$), and $Q_T$ = the total charge passed during the CPE experiment in Coulombs.

Randles-Sevcik equation:

$i_p = 0.496(FAC)\sqrt{(DF\nu/RT)}$

where $i_p$ = peak current observed in CV in Amperes, F = Faraday's constant, A = surface area of the electrode in $cm^2$, C = concentration of electroactive species in $mol/cm^3$, D = diffusion

coefficient of the electroactive species in $cm^2/s$, ν = the scan rate in V/s, R = ideal gas constant, and T = absolute temperature. The Randles-Sevcik equation describes the peak current of a mass-transport limited event and establishes the relationship of peak current being proportional to the square root of the scan rate as evidence for homogeneity.[9,10]

Cottrell equation:

$i(t) = (nFAD_O^{1/2}\ C^*{}_O^{1/2})/\sqrt{}(\pi^{1/2}t^{1/2})$

where i(t) = current in Amperes, n = number of electrons, F =Faraday's constant, A = surface area of the electrode in $cm^2$, $D_O$ = diffusion coefficient of the electroactive species in $cm^2/s$, $C^*{}_O$ = initial concentration of the electroactive species in $mol/cm^3$, and t = time in seconds. The Cottrell equation describes the current-time relationship during diffusion-limited evens, which affects electrolytic rate.[9,10]

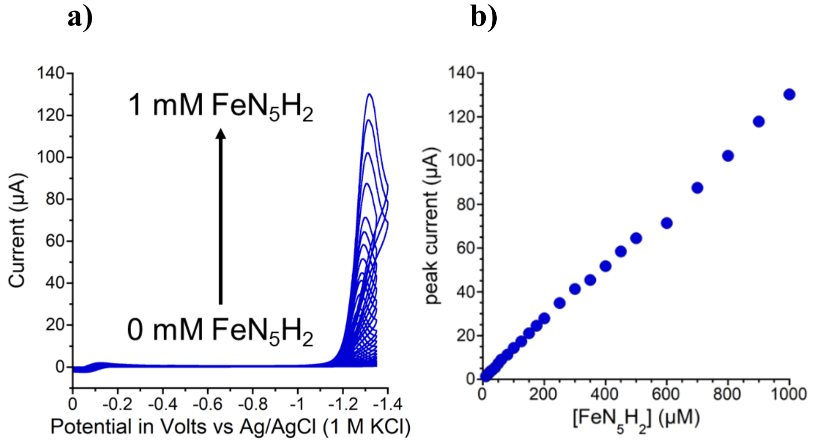


**Figure S7.** a) CVs (100 mV/s) of 1.0 M $KNO_3$ and 1.0 M MOPS at pH 7.2 as $FeN_5H_2$ is titrated in from 0 to 1 mM. b) A plot of peak current vs $[FeN_5H_2]$ shows linearity consistent with homogeneity.

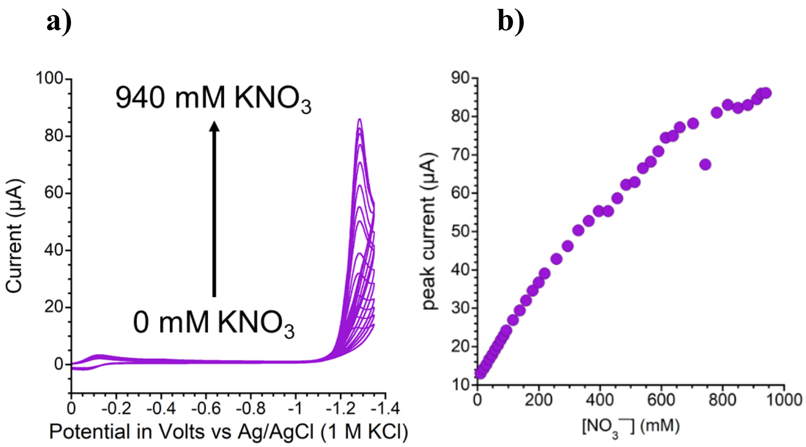


**Figure S8.** a) CVs (100 mV/s) of 1.0 M MOPS and 500 μM $FeN_5H_2$ at pH 7.2 and as $KNO_3$ is titrated in from 0 to 940 mM. b) A plot of peak current vs [$KNO_3$] showing linearity at low [$KNO_3$] consistent with homogeneity.

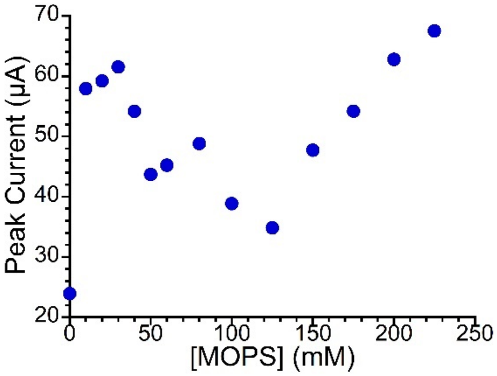


**Figure S9**. Plot of CV peak current vs [MOPS] from CVs (100 mV/s) of 1.0 M $KNO_3$ and 500 μM $FeN_5H_2$ at pH 7.2.

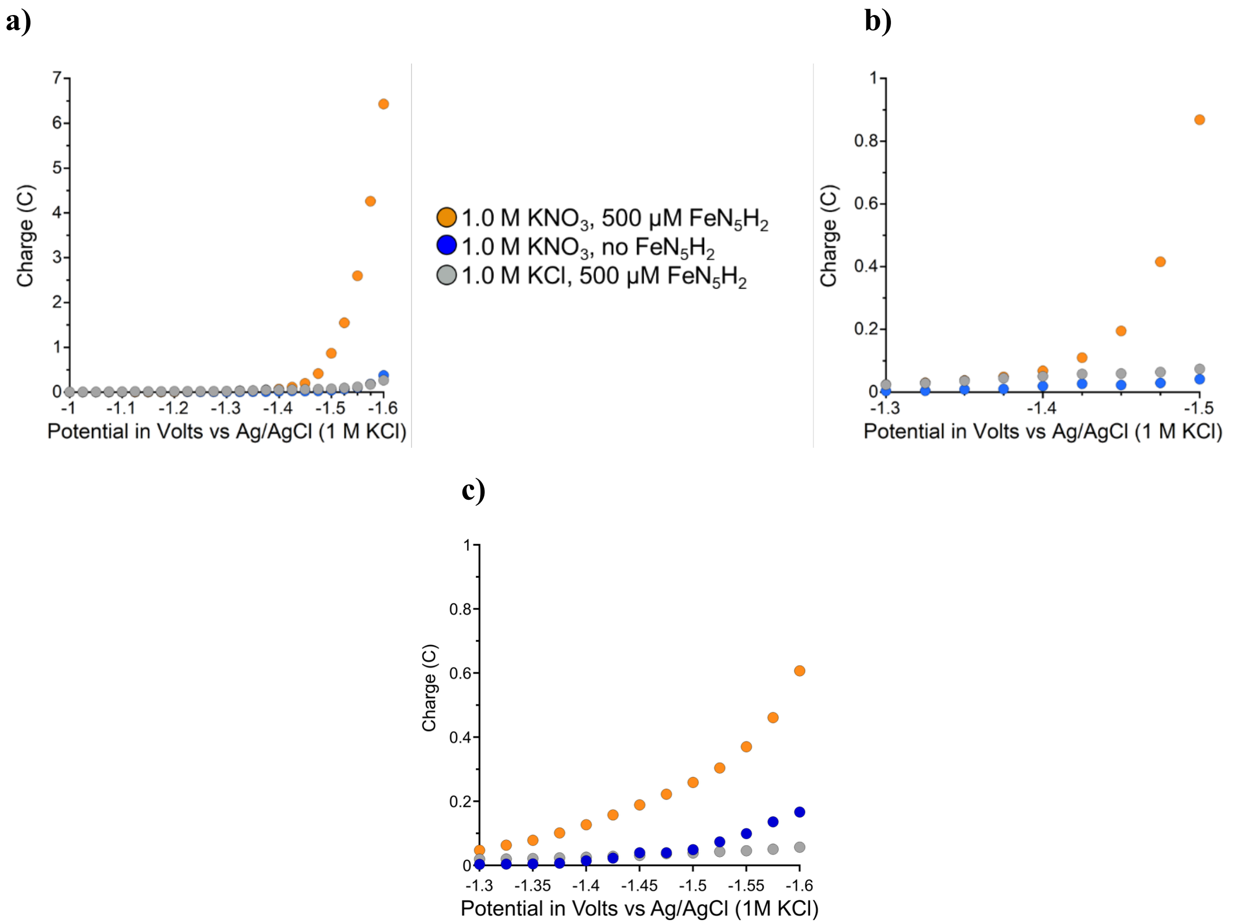


**Figure S10.** a) Charge passed after 1.0 min CPEs of a solution containing 1.0 M MOPS at pH 7.2, $KNO_3$ or KCl and $\mathbf{FeN_5H_2}$ as indicated in the legend. b) Magnification of the y-axis for the -1.3 to -1.5 V vs Ag/AgCl (1.0 M KCl) region. c) Charge passed after 1.0 min CPEs of a solution containing 225 mM MOPS at pH 7.2, 1.0 M $KNO_3$ or KCl, and $\mathbf{FeN_5H_2}$ as indicated in the legend.

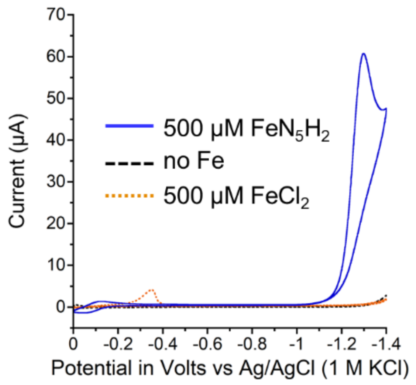


**Figure S11.** CV (100 mV/s) of 1.0 M $KNO_3$ and 1.0 M MOPS at pH 7.2 with $FeCl_2$ or $\mathbf{FeN_5H_2}$ as indicated in legend.

## Product Quantification

With respect to each product's degradation time and the time taken to perform the quantification, the following were run in the given order following CPE, with the exception of the Mb test, during which only determinations for NO were performed.

Ammonium is quantified by the indophenol blue test.[11] A calibration curve for the test is shown in Fig. S13. $^{14}N$ NMR was used to confirm ammonium production (Fig. S14).

Gas chromatography with a thermal conductivity detector (GC-TCD) is utilized for quantification of $N_2O$ and $H_2$. For analysis of $N_2O$, A Shimadzu GC-2014 equipped with an Agilent HP-PLOT Q column and a thermal conductivity detector (TCD) was used. Helium was used as a carrier gas for an isothermal 6-minute run at 30 ˚C. For $N_2O$, the peak area of a 100 µL injection from the headspace of a CPE experiment is compared to a calibration curve of known standards made from AA grade $N_2O$ (Airgas) (Figure S15a). For analysis of $H_2$, a Shimadzu GC-2014 equipped with a Restek RT-Msieve 5 Å column and a TCD was used with $N_2$ as a carrier gas. The samples were purged with an 80:20 $N_2/CH_4$ mixture (Airgas) prior to running CPE, with $CH_4$ serving as an internal standard. For $H_2$, the peak area of a 25 µL injection from the headspace of a CPE experiment is compared to a calibration curve of known standards made from AA grade $H_2$ (Airgas) (Figure S15b). GC-TCD chromatographs are shown for the CPE experiments (Figures S16 and S17). The calibration curve is used to obtain volume of $N_2O$ or $H_2$ present. The ideal gas law (PV = nRT) is utilized to calculate moles of $N_2O$ or $H_2$ using P = 0.98 atm, R = 0.082057 (L×atm)/(mol×K), and T = 293 K. Note that $H_2$ determinations were made in the absence of $KNO_3$ because products of $NO_3^-$ reduction (NO, $N_2O$, $NO_2$) are incompatible with the column used for $H_2$ determinations.

Hydroxylamine is quantified by redox titration of 20 mL 25 wt% NaOH mixed with 1-3 mL of reaction mixture from the CPE experiments with 5.0 mM $Fe^{III}(CN)_6$ to a yellow endpoint as indicated by:[12]

(1) $2\ NH_2OH + 4\ [Fe(CN)_6]^{3-} \rightarrow 4\ [Fe(CN)_6]^{4-} + N_2O + 4\ H^+ + H_2O$

Nitrite is quantified by the Griess test. A calibration curve for the test is shown in Fig. S18.

A myoglobin (Mb) test is performed to detect NO, a suspected intermediate given the reactivity of nitrite reductases.[13] A CPE was performed for 24 h (1.0 M $KNO_3$, 1.0 M MOPS at pH 7.2, and 500 μM $FeN_5H_2$ at -1.5 V vs Ag/AgCl (1.0 M KCl). Flowing $N_2$ gas was bubbling through the headspace of the CPE cell containing a solution of 500 μM Fe(II) Mb (Mb(II)) in 100 mM phosphate buffer at pH 7.4. Prior to, during, and following CPE, a 50x dilution of the Mb(II) solution was assessed by absorption spectroscopy. No shift in Soret or Q bands of Mb was observed (Fig. S19). This experiment does not detect NO as a product from CPE experiments.

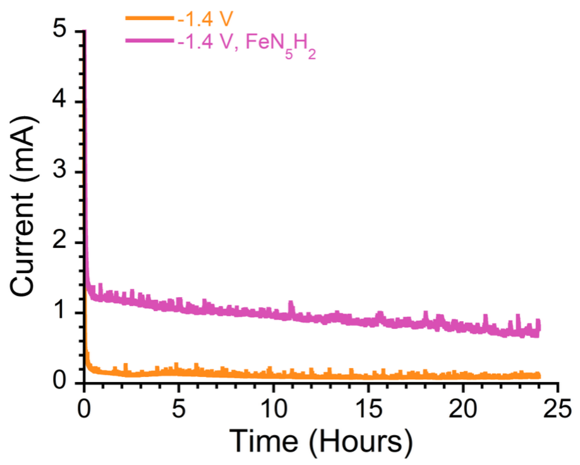


**Figure S12.** Plot of current vs. time for the CPE experiment at −1.4 V shown in Fig. 3 of the main text.

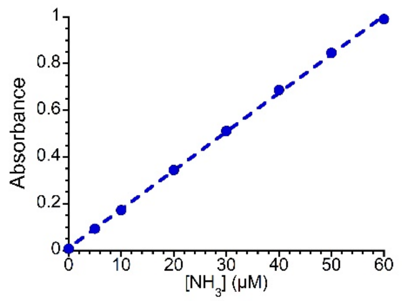


**Figure S13.** Indophenol blue test calibration curve made by plotting the absorbance at 650 nm of each standard versus the ammonia concentration of that standard. The best fit line has an equation of y = 0.0166x + 0.0112 with $R^2$ = 0.9995.

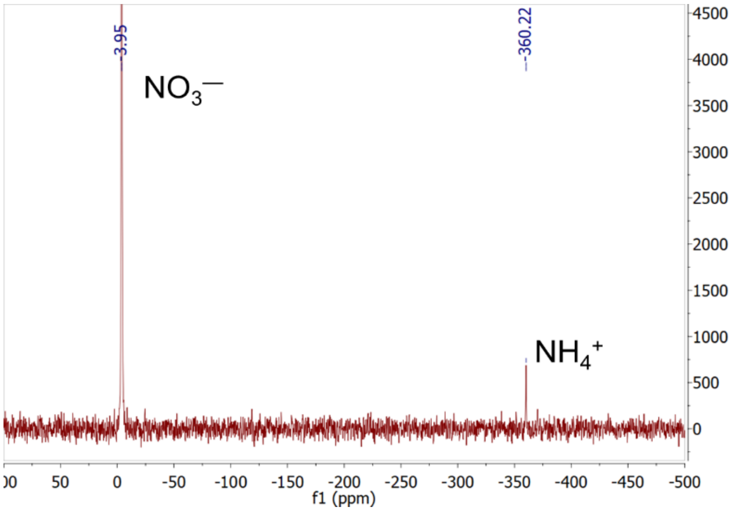


**Figure S14.** 36.1 MHz $^{14}N$ NMR of the solution from a CPE experiment run at −1.5 V in the presence of 1.0 M MOPS, 1.0 M $KNO_3$, and 500 μM $FeN_5H_2$ at pH 7.2 over 24 h confirming formation of $NH_4^+$.

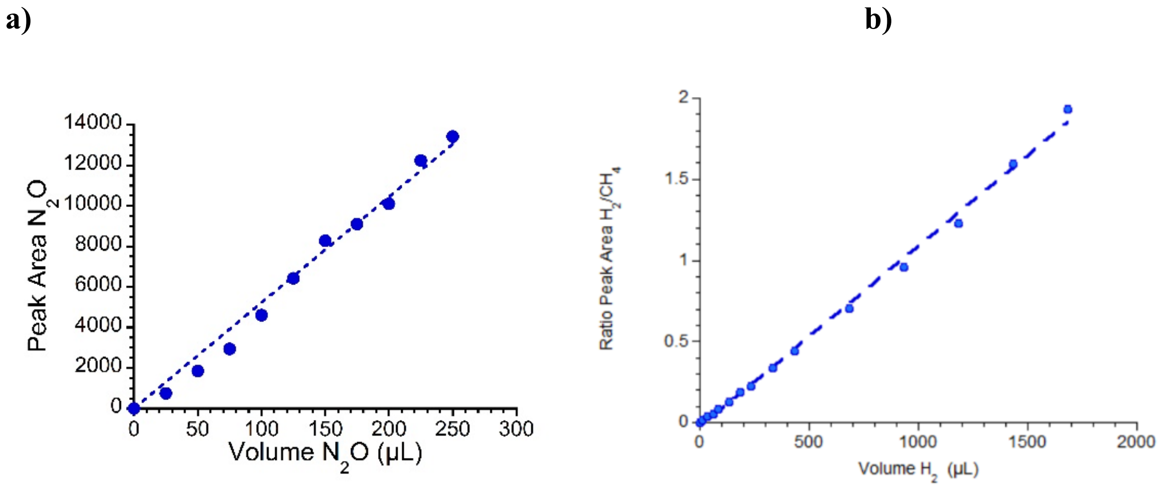


**Figure S15.** a) GC-TCD calibration curve for $N_2O$ was made by measuring peak area of 100 µL injections of $N_2O$ standards. The best fit line has an equation of y = 52.196x with $R^2 = 0.9956$. b) GC-TCD calibration curve for $H_2$ was made by measuring peak area of 25 µL injections of $H_2/CH_4$ standards. The best fit line has an equation of y = 0.0011x with $R^2 = 0.9977$.

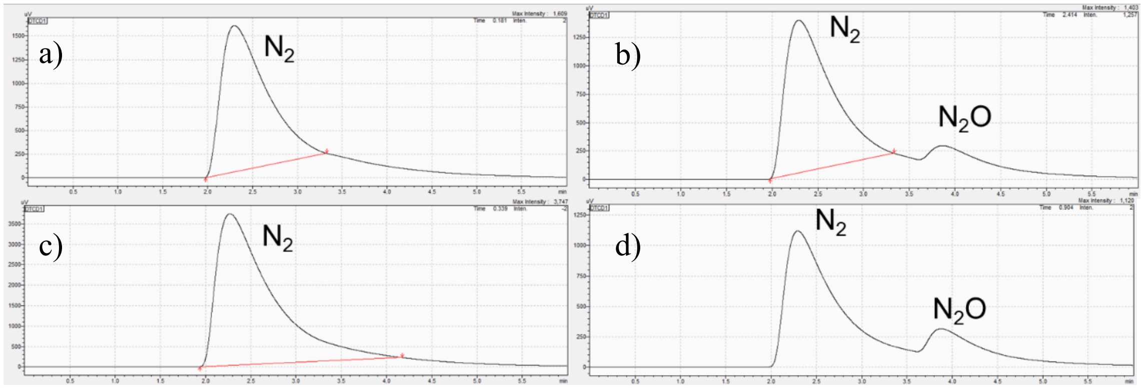


**Figure S16.** GC-TCD chromatographs for the CPE experiments containing 1.0 M $KNO_3$ and 1.0 M MOPS at pH 7.2 over 24 h with helium headspace. a) no $FeN_5H_2$ added at -1.4 V. b) 500 µM $FeN_5H_2$ at -1.4 V. c) no $FeN_5H_2$ added at -1.5 V. d) 500 µM $FeN_5H_2$ added at -1.5 V.

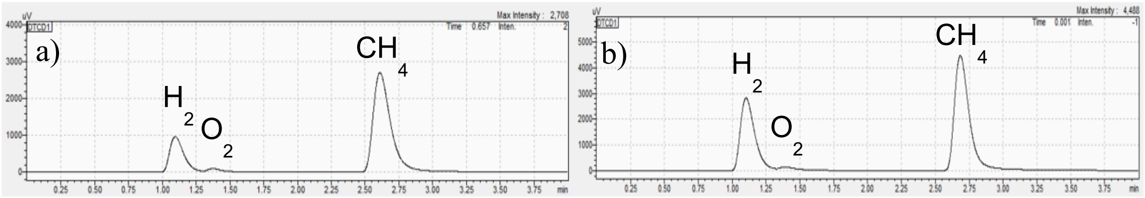


**Figure S17.** GC-TCD chromatographs for the CPE experiments containing 1.0 M KCl and 1.0 M MOPS at pH 7.2 over 2 h with $CH_4/N_2$ headspace. a) no $FeN_5H_2$ added at -1.5 V. b) 500 µM $FeN_5H_2$ at -1.5 V.

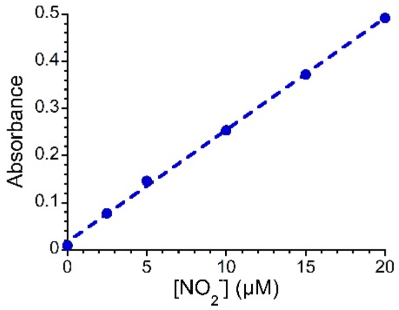


**Figure S18.** Griess test calibration curve made by plotting the absorbance at 548 nm of each standard versus the nitrite concentration of that standard. The best fit line has an equation of $y = 0.0238x + 0.0169$

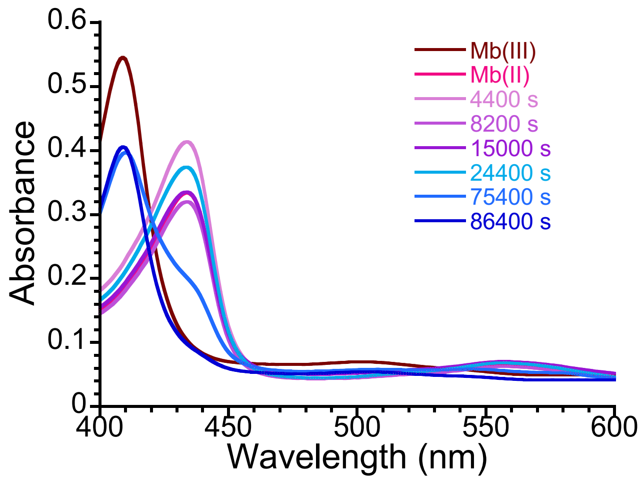


**Figure S19.** UV-vis spectra of 50x dilutions of 500 µM Mb in 100 mM phosphate buffer at pH 7.4 solution during the Mb test for NO. Mb(II) spectrum was taken prior to the start of the CPE while the solution was in the presence of oxygen. Then, 40 M sodium dithionite was added to solution prior to the start of CPE under flowing $N_2$, and the Mb(III) spectrum was observed as shown. The 24 h CPE was performed with removal of Mb solution to take spectra until the end of CPE. No peak indicative of Mb-NO adduct formation was observed.

**Table S1.** Limits of detection (LOD) for the nitrate reduction products of the CPE experiments. Calculations are based on duplicate calibration curve experiments using the standard deviations with respect to the *y*-intercepts.

| **Product** | **LOD** |
|---|---|
| $NO_2^-$ | 3 µM |
| $NH_2OH$ | 0.4 mM |
| $NH_4^+$ | 1 µM |
| $N_2O$ | 60 µL |
| $H_2$ | 20 µL |

**Table S2.** Summary of data collected from the 24-hour CPE experiments at −1.4 and −1.5 V vs Ag/AgCl (1.0 M KCl). Standard errors are given for duplicate experiments.

| Parameter | -1.4 V no $FeN_5H_2$ | -1.4 V 500 µM $FeN_5H_2$ | -1.5 V no $FeN_5H_2$ | -1.5 V 500 µM $FeN_5H_2$ |
|---|---|---|---|---|
| total charge (C) | 10 ± 3 | 83 ± 1 | 49.2 ± 2 | 185 ± 8 |
| starting pH | 7.2 | 7.2 | 7.2 | 7.2 |
| final pH | 7.2 | 7.7 | 7.5 | 8.4 |
| FE for $NO_2^-$ | 2.2 ± 0.2 % | 0.20 ± 0.03 % | 3.3 ± 0.5 % | 0.9 ± 0.7 % |
| µmol $NO_2^-$ | 6 ± 5 | 1.3 ± 0.5 | 7 ± 1 | 1.7 ± 0.5 |
| TON for $NO_2^-$ | NA[a] | 0.5 ± 0.2 | N/A | 0.6 ± .1 |
| FE for $NH_2OH$ | ND[b] | 15 ± 3 % | 14 ± 1 % | 26 ± 1 % |
| µmol $NH_2OH$ | ND | 22 ± 7 | 10 ± 3 | 44 ± 3 |
| TON for $NH_2OH$ | NA | 9 ± 2 | N/A | 17 ± 1 |
| FE for $NH_4^+$ | ND | 40 ± 1 % | 20 ± 2% | 33 ± 4 % |
| µmol $NH_4^+$ | ND | 42 ± 1 | ND | 79 ± 6 |
| TON for $NH_4^+$ | NA | 17 ± 1 | N/A | 27 ± 6 |
| FE for $N_2O$ | ND | 2.7 ± 0.4 % | ND | 2.3 ± 0.9 % |
| µmol $N_2O$ | ND | 3.3 ± 0.1 | ND | 5.7 ± 0.6 |
| TON for $N_2O$ | NA | 1.1 ± 0.2 | NA | 2.3 ± 1.6 |

[a]NA = not applicable

[b]ND = not detected

**Table S3.** Summary of data collected from the 2- and 24-hour CPE experiments at −1.3 V vs Ag/AgCl (1.0 M KCl). Standard errors are given for duplicate experiments.

| Parameter | -1.3 V, 2 h no $FeN_5H_2$ | -1.3 V, 2 h 500 µM $FeN_5H_2$ | -1.3 V, 24 h no $FeN_5H_2$ | -1.3 V, 24 h 500 µM $FeN_5H_2$ |
|---|---|---|---|---|
| **total charge (C)** | 0.4 ± 0.1 | 2.0 ± 1.1 | 5.2 ± 3.1 | 16.9 ± 7.8 |
| **starting pH** | 7.2 | 7.2 | 7.2 | 7.2 |
| **final pH** | 7.3 | 7.3 | 7.3 | 7.4 |
| **FE for $NO_2^-$** | 3.2 ± 3% | 3.9 ± 1.2% | 6.7 ± 1.1% | 1.8 ± 1.4% |
| **µmol $NO_2^-$** | 0.1 ± 0.1 | 0.4 ± 0.1 | 2.0 ± 1.4 | 1.0 ± 0.5 |
| **TON for $NO_2^-$** | NA[a] | 0.1 ± 0.02 | NA | 0.4 ± 0.2 |
| **FE for $NH_2OH$** | ND[b] | ND | ND | ND |
| **µmol $NH_2OH$** | ND | ND | ND | ND |
| **TON for $NH_2OH$** | N/A | ND | N/A | ND |
| **FE for $NH_4^+$** | ND | ND | ND | ND |
| **µmol $NH_4^+$** | ND | ND | ND | ND |
| **TON for $NH_4^+$** | NA | NA | NA | ND |
| **FE for $N_2O$** | ND | ND | ND | ND |
| **µmol $N_2O$** | ND | ND | ND | ND |
| **TON for $N_2O$** | NA | ND | NA | ND |

[a]NA = not applicable
[b]ND = not detected

**Table S4.** Summary of data collected from the 1-min nitrate reduction CPE experiments at −1.5 V vs Ag/AgCl (1.0 M KCl). Standard deviations are given for duplicate experiments.

| Parameter | -1.5 V, 1 min. no $FeN_5H_2$ | -1.5 V, 1 min. 500 µM $FeN_5H_2$ |
|---|---|---|
| **total charge (C)** | 0.3 ± 0.05 | 1.0 ± 0.07 |
| **starting pH** | 7.2 | 7.2 |
| **final pH** | 7.3 | 7.3 |
| **FE for $NO_2^-$** | ND | 2.3 ± 0.4% |
| **µmol $NO_2^-$** | ND | 0.1 ± 0.03 |
| **TON for $NO_2^-$** | N/A | 0.05 ± 0.01 |

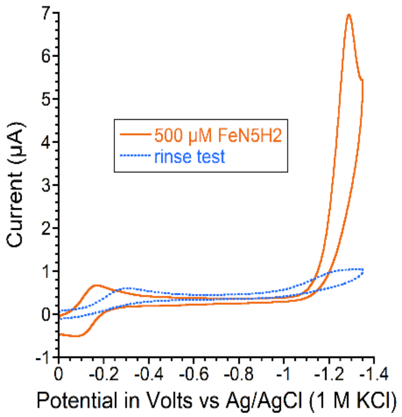


**Figure S20.** CV (100 mV/s) of 1.0 M $KNO_3$, 1.0 M MOPS at pH 7.2, and 500 µM $FeN_5H_2$ (orange, solid trace). Then, in a modified rinse test, the same mercury drop is re-immersed in a solution containing only 1.0 M $KNO_3$ and 1.0 M MOPS at pH 7.2 with no $FeN_5H_2$ and CV is repeated. No activity is observed in the rinse test. An oxygen peak was detected in the $Fe^{III/II}$ region due to the difficulty of maintaining the mercury drop while purging with $N_2$.

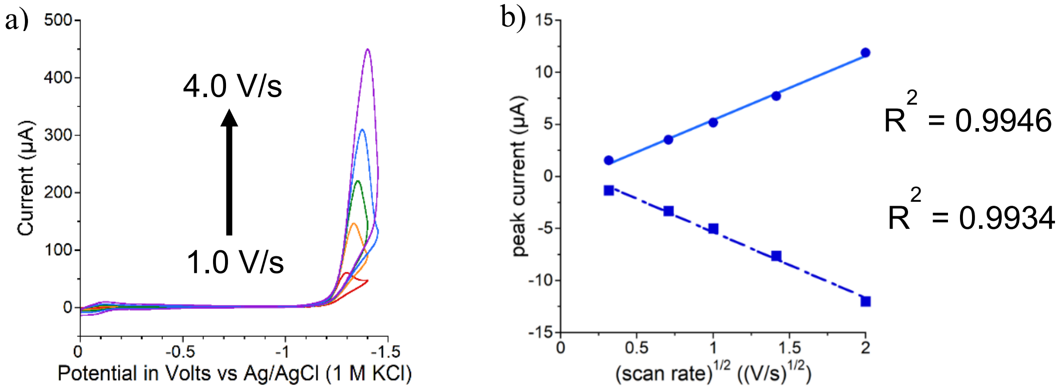


**Figure S21.** a) Cyclic voltammograms (CVs) of 1.0 M $KNO_3$, 1.0 M MOPS, and 500 µM $FeN_5H_2$ at pH 7.2 at scan rates ranging from 0.1 to 4 V/s. b) A plot of peak current versus scan rate for the $Fe^{III/II}$ event at -0.10 V vs Ag/AgCl (1.0 M KCl) demonstrating homogeneity. The best fit lines have equations of $y = 6.1594x – 0.72041$ with $R^2 = 0.99457$ and $y = -6.3564x +1.0606$ with $R^2 = 0.99338$.

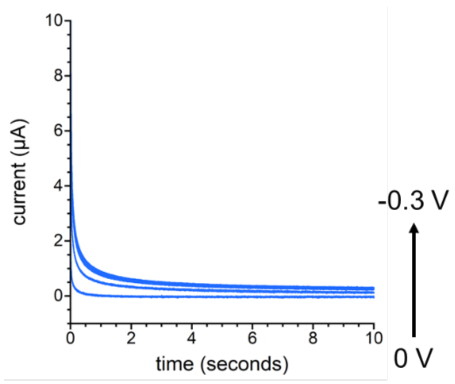


**Figure S22.** Chronoamperograms of 1.0 M $KNO_3$ and 1.0 M MOPS at pH 7.2 with $FeN_5H_2$ as indicated in the legend. Potential is stepped from 0 to -0.3 V vs Ag/AgCl (1.0 M KCl) in 25-mV increments.

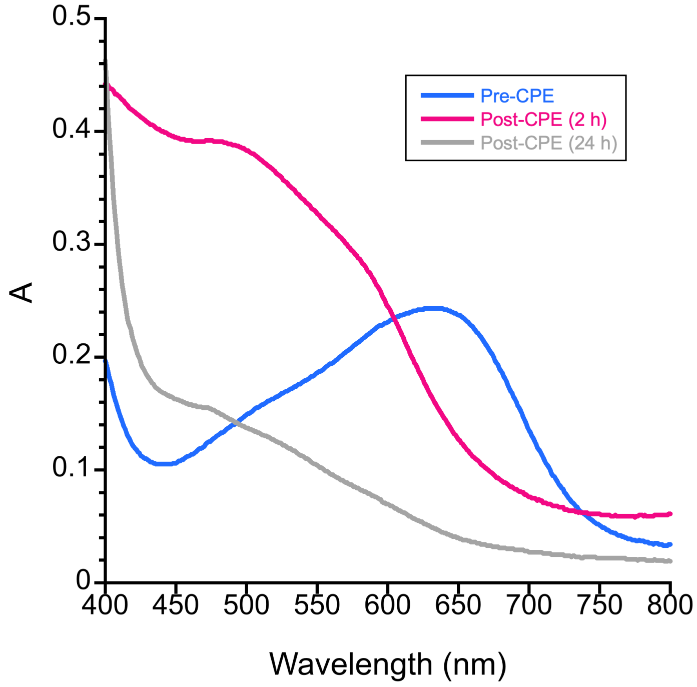


**Figure S23.** UV-vis spectra of 500 mM $Fe(II)N_5H_2$, 1 M $KNO_3$, 1 M MOPS, pH 7.2 before electrolysis and after 2 or 24 hours.

**Table S4.** Crystal data and structure refinement for **$FeN_5H_2$**.

| | | |
|---|---|---|
| Empirical formula | C15 H27 Cl2 Fe N5 O6 | |
| Formula weight | 500.16 | |
| Temperature | 100.00(10) K | |
| Wavelength | 1.54184 Å | |
| Crystal system | orthorhombic | |
| Space group | *Pbca* | |
| Unit cell dimensions | *a* = 8.12520(10) Å | a = 90° |
| | *b* = 19.3003(2) Å | b = 90° |
| | *c* = 26.7777(3) Å | g = 90° |
| Volume | 4199.25(8) $Å^3$ | |
| *Z* | 8 | |
| Density (calculated) | 1.582 $Mg/m^3$ | |
| Absorption coefficient | 8.484 $mm^{-1}$ | |
| *F*(000) | 2080 | |
| Crystal color, morphology | dark blue, plate | |
| Crystal size | 0.154 x 0.128 x 0.056 $mm^3$ | |
| Theta range for data collection | 3.301 to 80.374° | |
| Index ranges | -7 £ *h* £ 10, -24 £ *k* £ 24, -33 £ *l* £ 34 | |
| Reflections collected | 37770 | |
| Independent reflections | 4545 [*R*(int) = 0.0668] | |
| Observed reflections | 4118 | |
| Completeness to theta = 74.504° | 99.8% | |
| Absorption correction | Multi-scan | |
| Max. and min. transmission | 1.00000 and 0.16319 | |
| Refinement method | Full-matrix least-squares on $F^2$ | |
| Data / restraints / parameters | 4545 / 144 / 334 | |
| Goodness-of-fit on $F^2$ | 1.049 | |
| Final *R* indices [*I*>2sigma(*I*)] | *R*1 = 0.0376, *wR*2 = 0.1011 | |
| *R* indices (all data) | *R*1 = 0.0415, *wR*2 = 0.1043 | |
| Largest diff. peak and hole | 0.484 and -0.444 e.$Å^{-3}$ | |

**Table S5.** Atomic coordinates (x $10^4$) and equivalent isotropic displacement parameters ($Å^2$x $10^3$) for $FeN_5H_2$. $U_{eq}$ is defined as one third of the trace of the orthogonalized $U_{ij}$ tensor.

| | x | y | z | $U_{eq}$ |
|---|---|---|---|---|
| Fe1 | 6845(1) | 3735(1) | 3895(1) | 12(1) |
| O1 | 4677(2) | 4379(1) | 3944(1) | 22(1) |
| O2 | 9050(2) | 3096(1) | 3820(1) | 21(1) |
| N1 | 6943(2) | 3873(1) | 3068(1) | 15(1) |
| N2 | 5512(2) | 2854(1) | 3513(1) | 16(1) |
| N3 | 5738(2) | 3055(1) | 4511(1) | 16(1) |
| N4 | 7895(2) | 4157(1) | 4619(1) | 17(1) |
| N5 | 8223(2) | 4706(1) | 3707(1) | 17(1) |
| C1 | 6248(2) | 3401(1) | 2768(1) | 16(1) |
| C2 | 6318(3) | 3458(1) | 2250(1) | 21(1) |
| C3 | 7143(3) | 4018(1) | 2044(1) | 24(1) |
| C4 | 7871(3) | 4507(1) | 2355(1) | 22(1) |
| C5 | 7746(2) | 4415(1) | 2872(1) | 17(1) |
| C6 | 5411(2) | 2821(1) | 3038(1) | 16(1) |
| C7 | 4586(3) | 2264(1) | 2737(1) | 22(1) |
| C8 | 8495(2) | 4882(1) | 3254(1) | 18(1) |
| C9 | 9462(3) | 5506(1) | 3096(1) | 26(1) |
| C10 | 4763(3) | 2329(1) | 3836(1) | 20(1) |
| C11 | 5663(2) | 2336(1) | 4332(1) | 18(1) |
| C12 | 6641(3) | 3139(1) | 4981(1) | 21(1) |
| C13 | 6983(3) | 3900(1) | 5057(1) | 21(1) |
| C14 | 7980(3) | 4916(1) | 4592(1) | 21(1) |
| C15 | 8881(3) | 5116(1) | 4120(1) | 22(1) |
| Cl1 | 1839(1) | 3657(1) | 4475(1) | 20(1) |
| Cl2 | 9903(4) | 1250(2) | 3506(1) | 16(1) |
| O3 | 8471(3) | 1697(1) | 3540(1) | 31(1) |
| O4 | 11338(5) | 1670(2) | 3455(2) | 46(1) |
| O5 | 9702(7) | 799(3) | 3089(2) | 27(1) |
| O6 | 9971(4) | 850(1) | 3961(1) | 42(1) |
| Cl2' | 10176(12) | 1228(5) | 3516(4) | 24(2) |

| | | | | |
|---|---|---|---|---|
| O3' | 8965(12) | 1380(6) | 3875(3) | 65(3) |
| O4' | 11027(17) | 1844(6) | 3391(7) | 45(3) |
| O5' | 9372(19) | 953(8) | 3079(5) | 34(3) |
| O6' | 11339(9) | 735(3) | 3697(3) | 41(2) |

**Table S6.** Bond lengths [Å] and angles [°] for $FeN_5H_2$.

| | | | |
|---|---|---|---|
| Fe(1)-O(1) | 2.1602(15) | C(4)-H(4A) | 0.9500 |
| Fe(1)-O(2) | 2.1850(15) | C(4)-C(5) | 1.402(3) |
| Fe(1)-N(1) | 2.2325(17) | C(5)-C(8) | 1.493(3) |
| Fe(1)-N(2) | 2.2608(16) | C(6)-C(7) | 1.503(3) |
| Fe(1)-N(3) | 2.2932(16) | C(7)-H(7A) | 0.9800 |
| Fe(1)-N(4) | 2.2695(17) | C(7)-H(7B) | 0.9800 |
| Fe(1)-N(5) | 2.2406(17) | C(7)-H(7C) | 0.9800 |
| O(1)-H(1A) | 0.90(4) | C(8)-C(9) | 1.498(3) |
| O(1)-H(1B) | 0.82(3) | C(9)-H(9A) | 0.9800 |
| O(2)-H(2A) | 0.76(3) | C(9)-H(9B) | 0.9800 |
| O(2)-H(2B) | 0.88(3) | C(9)-H(9C) | 0.9800 |
| N(1)-C(1) | 1.339(3) | C(10)-H(10A) | 0.9900 |
| N(1)-C(5) | 1.338(3) | C(10)-H(10B) | 0.9900 |
| N(2)-C(6) | 1.276(3) | C(10)-C(11) | 1.517(3) |
| N(2)-C(10) | 1.463(2) | C(11)-H(11A) | 0.9900 |
| N(3)-H(3) | 0.92(3) | C(11)-H(11B) | 0.9900 |
| N(3)-C(11) | 1.469(3) | C(12)-H(12A) | 0.9900 |
| N(3)-C(12) | 1.466(3) | C(12)-H(12B) | 0.9900 |
| N(4)-H(4) | 0.86(3) | C(12)-C(13) | 1.508(3) |
| N(4)-C(13) | 1.473(3) | C(13)-H(13A) | 0.9900 |
| N(4)-C(14) | 1.469(3) | C(13)-H(13B) | 0.9900 |
| N(5)-C(8) | 1.280(3) | C(14)-H(14A) | 0.9900 |
| N(5)-C(15) | 1.461(3) | C(14)-H(14B) | 0.9900 |
| C(1)-C(2) | 1.393(3) | C(14)-C(15) | 1.512(3) |
| C(1)-C(6) | 1.495(3) | C(15)-H(15A) | 0.9900 |
| C(2)-H(2) | 0.9500 | C(15)-H(15B) | 0.9900 |
| C(2)-C(3) | 1.386(3) | Cl(2)-O(3) | 1.451(4) |
| C(3)-H(3A) | 0.9500 | Cl(2)-O(4) | 1.428(4) |
| C(3)-C(4) | 1.389(3) | Cl(2)-O(5) | 1.426(4) |

| | | | |
|---|---|---|---|
| Cl(2)-O(6) | 1.442(4) | C(6)-N(2)-C(10) | 121.87(17) |
| Cl(2')-O(3') | 1.408(11) | C(10)-N(2)-Fe(1) | 116.92(12) |
| Cl(2')-O(4') | 1.416(12) | Fe(1)-N(3)-H(3) | 107.9(18) |
| Cl(2')-O(5') | 1.441(11) | C(11)-N(3)-Fe(1) | 108.76(11) |
| Cl(2')-O(6') | 1.425(10) | C(11)-N(3)-H(3) | 109.1(18) |
| O(1)-Fe(1)-O(2) | 178.13(6) | C(12)-N(3)-Fe(1) | 111.01(12) |
| O(1)-Fe(1)-N(1) | 91.22(6) | C(12)-N(3)-H(3) | 105.9(18) |
| O(1)-Fe(1)-N(2) | 94.04(6) | C(12)-N(3)-C(11) | 113.96(16) |
| O(1)-Fe(1)-N(3) | 88.03(6) | Fe(1)-N(4)-H(4) | 107(2) |
| O(1)-Fe(1)-N(4) | 92.73(6) | C(13)-N(4)-Fe(1) | 111.80(12) |
| O(1)-Fe(1)-N(5) | 86.55(6) | C(13)-N(4)-H(4) | 106(2) |
| O(2)-Fe(1)-N(1) | 86.96(6) | C(14)-N(4)-Fe(1) | 109.48(12) |
| O(2)-Fe(1)-N(2) | 85.78(6) | C(14)-N(4)-H(4) | 108(2) |
| O(2)-Fe(1)-N(3) | 93.69(6) | C(14)-N(4)-C(13) | 113.46(16) |
| O(2)-Fe(1)-N(4) | 88.43(6) | C(8)-N(5)-Fe(1) | 121.46(14) |
| O(2)-Fe(1)-N(5) | 92.40(6) | C(8)-N(5)-C(15) | 120.69(17) |
| N(1)-Fe(1)-N(2) | 70.02(6) | C(15)-N(5)-Fe(1) | 117.78(13) |
| N(1)-Fe(1)-N(3) | 142.81(6) | N(1)-C(1)-C(2) | 121.67(19) |
| N(1)-Fe(1)-N(4) | 142.35(6) | N(1)-C(1)-C(6) | 114.30(17) |
| N(1)-Fe(1)-N(5) | 70.09(6) | C(2)-C(1)-C(6) | 124.03(19) |
| N(2)-Fe(1)-N(3) | 72.95(6) | C(1)-C(2)-H(2) | 120.8 |
| N(2)-Fe(1)-N(4) | 146.72(6) | C(3)-C(2)-C(1) | 118.5(2) |
| N(4)-Fe(1)-N(3) | 74.77(6) | C(3)-C(2)-H(2) | 120.8 |
| N(5)-Fe(1)-N(2) | 140.11(6) | C(2)-C(3)-H(3A) | 120.0 |
| N(5)-Fe(1)-N(3) | 146.79(6) | C(2)-C(3)-C(4) | 119.91(19) |
| N(5)-Fe(1)-N(4) | 72.80(6) | C(4)-C(3)-H(3A) | 120.0 |
| Fe(1)-O(1)-H(1A) | 119(2) | C(3)-C(4)-H(4A) | 120.9 |
| Fe(1)-O(1)-H(1B) | 127(2) | C(3)-C(4)-C(5) | 118.3(2) |
| H(1A)-O(1)-H(1B) | 109(3) | C(5)-C(4)-H(4A) | 120.9 |
| Fe(1)-O(2)-H(2A) | 125(2) | N(1)-C(5)-C(4) | 121.40(19) |
| Fe(1)-O(2)-H(2B) | 120(2) | N(1)-C(5)-C(8) | 113.84(17) |
| H(2A)-O(2)-H(2B) | 107(3) | C(4)-C(5)-C(8) | 124.74(18) |
| C(1)-N(1)-Fe(1) | 119.85(13) | N(2)-C(6)-C(1) | 114.59(17) |
| C(5)-N(1)-Fe(1) | 119.87(14) | N(2)-C(6)-C(7) | 126.79(19) |
| C(5)-N(1)-C(1) | 120.24(17) | C(1)-C(6)-C(7) | 118.60(17) |
| C(6)-N(2)-Fe(1) | 121.21(14) | C(6)-C(7)-H(7A) | 109.5 |

| | |
|---|---|
| C(6)-C(7)-H(7B) | 109.5 |
| C(6)-C(7)-H(7C) | 109.5 |
| H(7A)-C(7)-H(7B) | 109.5 |
| H(7A)-C(7)-H(7C) | 109.5 |
| H(7B)-C(7)-H(7C) | 109.5 |
| N(5)-C(8)-C(5) | 114.65(17) |
| N(5)-C(8)-C(9) | 124.8(2) |
| C(5)-C(8)-C(9) | 120.50(18) |
| C(8)-C(9)-H(9A) | 109.5 |
| C(8)-C(9)-H(9B) | 109.5 |
| C(8)-C(9)-H(9C) | 109.5 |
| H(9A)-C(9)-H(9B) | 109.5 |
| H(9A)-C(9)-H(9C) | 109.5 |
| H(9B)-C(9)-H(9C) | 109.5 |
| N(2)-C(10)-H(10A) | 110.1 |
| N(2)-C(10)-H(10B) | 110.1 |
| N(2)-C(10)-C(11) | 108.09(16) |
| H(10A)-C(10)-H(10B) | 108.4 |
| C(11)-C(10)-H(10A) | 110.1 |
| C(11)-C(10)-H(10B) | 110.1 |
| N(3)-C(11)-C(10) | 108.34(16) |
| N(3)-C(11)-H(11A) | 110.0 |
| N(3)-C(11)-H(11B) | 110.0 |
| C(10)-C(11)-H(11A) | 110.0 |
| C(10)-C(11)-H(11B) | 110.0 |
| H(11A)-C(11)-H(11B) | 108.4 |
| N(3)-C(12)-H(12A) | 110.0 |
| N(3)-C(12)-H(12B) | 110.0 |
| N(3)-C(12)-C(13) | 108.41(16) |
| H(12A)-C(12)-H(12B) | 108.4 |
| C(13)-C(12)-H(12A) | 110.0 |
| C(13)-C(12)-H(12B) | 110.0 |
| N(4)-C(13)-C(12) | 108.23(17) |
| N(4)-C(13)-H(13A) | 110.1 |
| N(4)-C(13)-H(13B) | 110.1 |
| C(12)-C(13)-H(13A) | 110.1 |
| C(12)-C(13)-H(13B) | 110.1 |
| H(13A)-C(13)-H(13B) | 108.4 |
| N(4)-C(14)-H(14A) | 110.0 |
| N(4)-C(14)-H(14B) | 110.0 |
| N(4)-C(14)-C(15) | 108.58(16) |
| H(14A)-C(14)-H(14B) | 108.4 |
| C(15)-C(14)-H(14A) | 110.0 |
| C(15)-C(14)-H(14B) | 110.0 |
| N(5)-C(15)-C(14) | 108.53(17) |
| N(5)-C(15)-H(15A) | 110.0 |
| N(5)-C(15)-H(15B) | 110.0 |
| C(14)-C(15)-H(15A) | 110.0 |
| C(14)-C(15)-H(15B) | 110.0 |
| H(15A)-C(15)-H(15B) | 108.4 |
| O(4)-Cl(2)-O(3) | 108.9(3) |
| O(4)-Cl(2)-O(6) | 110.8(4) |
| O(5)-Cl(2)-O(3) | 108.6(3) |
| O(5)-Cl(2)-O(4) | 111.4(4) |
| O(5)-Cl(2)-O(6) | 109.8(3) |
| O(6)-Cl(2)-O(3) | 107.2(3) |
| O(3')-Cl(2')-O(4') | 109.1(11) |
| O(3')-Cl(2')-O(5') | 108.4(10) |
| O(3')-Cl(2')-O(6') | 111.7(9) |
| O(4')-Cl(2')-O(5') | 109.9(12) |
| O(4')-Cl(2')-O(6') | 108.4(8) |
| O(6')-Cl(2')-O(5') | 109.4(9) |

**Table S7.** Anisotropic displacement parameters ($Å^2 x 10^3$) for $FeN_5H_2$. The anisotropic displacement factor exponent takes the form: $-2p^2[ h^2 a^{*2}U_{11} + ... + 2 h k a^* b^* U_{12} ]$

| | $U_{11}$ | $U_{22}$ | $U_{33}$ | $U_{23}$ | $U_{13}$ | $U_{12}$ |
|---|---|---|---|---|---|---|
| Fe1 | 13(1) | 14(1) | 10(1) | 0(1) | 0(1) | 0(1) |
| O1 | 17(1) | 16(1) | 34(1) | 4(1) | 2(1) | 2(1) |
| O2 | 17(1) | 17(1) | 28(1) | -5(1) | -3(1) | 3(1) |
| N1 | 14(1) | 17(1) | 14(1) | 1(1) | 1(1) | 3(1) |
| N2 | 16(1) | 14(1) | 18(1) | 1(1) | -1(1) | -1(1) |
| N3 | 16(1) | 18(1) | 15(1) | 2(1) | 1(1) | 3(1) |
| N4 | 16(1) | 20(1) | 14(1) | -2(1) | 0(1) | 2(1) |
| N5 | 17(1) | 13(1) | 21(1) | -2(1) | -1(1) | -1(1) |
| C1 | 15(1) | 19(1) | 14(1) | -1(1) | -2(1) | 5(1) |
| C2 | 20(1) | 28(1) | 15(1) | -2(1) | -1(1) | 6(1) |
| C3 | 23(1) | 36(1) | 12(1) | 4(1) | 2(1) | 7(1) |
| C4 | 21(1) | 25(1) | 19(1) | 7(1) | 4(1) | 5(1) |
| C5 | 15(1) | 17(1) | 18(1) | 3(1) | 4(1) | 4(1) |
| C6 | 14(1) | 18(1) | 17(1) | -1(1) | -3(1) | 4(1) |
| C7 | 26(1) | 20(1) | 21(1) | -5(1) | -5(1) | -1(1) |
| C8 | 17(1) | 15(1) | 22(1) | 2(1) | 4(1) | 1(1) |
| C9 | 29(1) | 18(1) | 31(1) | 3(1) | 9(1) | -4(1) |
| C10 | 21(1) | 15(1) | 22(1) | 3(1) | 0(1) | -4(1) |
| C11 | 18(1) | 15(1) | 20(1) | 4(1) | 4(1) | 0(1) |
| C12 | 24(1) | 26(1) | 14(1) | 4(1) | -2(1) | 2(1) |
| C13 | 23(1) | 28(1) | 14(1) | -3(1) | 0(1) | 2(1) |
| C14 | 22(1) | 20(1) | 22(1) | -7(1) | -2(1) | 2(1) |
| C15 | 23(1) | 18(1) | 24(1) | -5(1) | -2(1) | -3(1) |
| Cl1 | 14(1) | 21(1) | 26(1) | -1(1) | 1(1) | 0(1) |
| Cl2 | 20(1) | 13(1) | 16(1) | 1(1) | 0(1) | -3(1) |
| O3 | 24(1) | 25(1) | 45(2) | -14(1) | 4(1) | 2(1) |
| O4 | 20(2) | 43(2) | 74(3) | 2(2) | -2(2) | -15(2) |
| O5 | 36(2) | 26(2) | 19(1) | -10(1) | 2(1) | 7(1) |
| O6 | 82(2) | 25(1) | 19(1) | 6(1) | -14(1) | -6(1) |
| Cl2' | 35(4) | 21(2) | 16(2) | -4(1) | -6(2) | 1(2) |

| | | | | | | |
|---|---|---|---|---|---|---|
| O3' | 55(5) | 102(7) | 40(4) | -26(4) | 19(4) | 7(4) |
| O4' | 48(5) | 31(4) | 58(6) | 13(4) | -27(4) | -10(4) |
| O5' | 32(6) | 43(7) | 26(3) | -9(4) | -5(3) | -3(5) |
| O6' | 41(3) | 22(3) | 60(5) | 5(3) | -24(3) | -3(2) |

**Table S8.** Hydrogen coordinates (x $10^4$) and isotropic displacement parameters (Å$^2$ x $10^3$) for $FeN_5H_2$.

| | x | y | z | U(eq) |
|---|---|---|---|---|
| H1A | 3800(50) | 4223(18) | 4113(13) | 50(9) |
| H1B | 4640(40) | 4801(18) | 3935(11) | 35(8) |
| H2A | 9050(40) | 2707(16) | 3778(10) | 25(7) |
| H2B | 9940(40) | 3192(16) | 3995(12) | 38(8) |
| H3 | 4680(40) | 3210(15) | 4573(11) | 33(8) |
| H4 | 8880(40) | 3998(16) | 4647(11) | 33(8) |
| H2 | 5813 | 3120 | 2042 | 25 |
| H3A | 7210 | 4067 | 1692 | 28 |
| H4A | 8439 | 4894 | 2219 | 26 |
| H7A | 5423 | 1954 | 2597 | 34 |
| H7B | 3955 | 2475 | 2465 | 34 |
| H7C | 3843 | 1998 | 2952 | 34 |
| H9A | 8775 | 5921 | 3129 | 39 |
| H9B | 9806 | 5453 | 2747 | 39 |
| H9C | 10436 | 5553 | 3309 | 39 |
| H10A | 4853 | 1866 | 3679 | 24 |
| H10B | 3583 | 2435 | 3887 | 24 |
| H11A | 5074 | 2043 | 4577 | 21 |
| H11B | 6790 | 2150 | 4290 | 21 |
| H12A | 7688 | 2878 | 4968 | 26 |
| H12B | 5978 | 2958 | 5263 | 26 |
| H13A | 5936 | 4157 | 5094 | 26 |
| H13B | 7644 | 3969 | 5364 | 26 |
| H14A | 8570 | 5100 | 4887 | 25 |
| H14B | 6856 | 5114 | 4589 | 25 |

| | | | | |
|---|---|---|---|---|
| H15A | 8726 | 5616 | 4051 | 26 |
| H15B | 10074 | 5026 | 4158 | 26 |

**Table S9.** Torsion angles [°] for $FeN_5H_2$.

| | | | |
|---|---|---|---|
| Fe1-N1-C1-C2 | -178.30(14) | C4-C5-C8-C9 | 0.2(3) |
| Fe1-N1-C1-C6 | 1.7(2) | C5-N1-C1-C2 | -0.7(3) |
| Fe1-N1-C5-C4 | 178.23(14) | C5-N1-C1-C6 | 179.32(16) |
| Fe1-N1-C5-C8 | -0.6(2) | C6-N2-C10-C11 | 156.57(18) |
| Fe1-N2-C6-C1 | 0.6(2) | C6-C1-C2-C3 | -179.53(18) |
| Fe1-N2-C6-C7 | 179.18(15) | C8-N5-C15-C14 | 163.83(18) |
| Fe1-N2-C10-C11 | -24.0(2) | C10-N2-C6-C1 | 179.96(16) |
| Fe1-N3-C11-C10 | -53.25(17) | C10-N2-C6-C7 | -1.4(3) |
| Fe1-N3-C12-C13 | 43.15(19) | C11-N3-C12-C13 | 166.35(16) |
| Fe1-N4-C13-C12 | 42.57(19) | C12-N3-C11-C10 | -177.67(16) |
| Fe1-N4-C14-C15 | -52.18(18) | C13-N4-C14-C15 | -177.83(16) |
| Fe1-N5-C8-C5 | 3.3(2) | C14-N4-C13-C12 | 166.98(16) |
| Fe1-N5-C8-C9 | -177.44(15) | C15-N5-C8-C5 | 180.00(17) |
| Fe1-N5-C15-C14 | -19.3(2) | C15-N5-C8-C9 | -0.7(3) |
| N1-C1-C2-C3 | 0.5(3) | | |
| N1-C1-C6-N2 | -1.4(2) | | |
| N1-C1-C6-C7 | 179.84(17) | | |
| N1-C5-C8-N5 | -1.7(2) | | |
| N1-C5-C8-C9 | 178.97(18) | | |
| N2-C10-C11-N3 | 50.4(2) | | |
| N3-C12-C13-N4 | -56.8(2) | | |
| N4-C14-C15-N5 | 46.2(2) | | |
| C1-N1-C5-C4 | 0.6(3) | | |
| C1-N1-C5-C8 | -178.21(16) | | |
| C1-C2-C3-C4 | -0.2(3) | | |
| C2-C1-C6-N2 | 178.53(19) | | |
| C2-C1-C6-C7 | -0.2(3) | | |
| C2-C3-C4-C5 | 0.1(3) | | |
| C3-C4-C5-N1 | -0.3(3) | | |
| C3-C4-C5-C8 | 178.36(18) | | |
| C4-C5-C8-N5 | 179.56(19) | | |

**Table S10.** Hydrogen bonds and close contacts for $FeN_5H_2$ [Å and °].

| D-H...A | d(D-H) | d(H...A) | d(D...A) | <(DHA) |
|---|---|---|---|---|
| O1-H1A...Cl1 | 0.90(4) | 2.16(4) | 3.0469(17) | 169(3) |
| O1-H1B...O6#1 | 0.82(3) | 2.05(3) | 2.853(3) | 168(3) |
| O1-H1B...O6'#1 | 0.82(3) | 2.07(3) | 2.823(7) | 153(3) |
| O2-H2A...O3 | 0.76(3) | 2.10(3) | 2.842(3) | 164(3) |
| O2-H2A...O3' | 0.76(3) | 2.57(3) | 3.315(12) | 166(3) |
| O2-H2A...O4' | 0.76(3) | 2.54(3) | 3.121(17) | 135(3) |
| O2-H2B...Cl1#2 | 0.88(3) | 2.20(3) | 3.0637(16) | 166(3) |
| N3-H3...Cl1 | 0.92(3) | 2.48(3) | 3.3762(17) | 164(2) |
| N4-H4...Cl1#2 | 0.86(3) | 2.54(3) | 3.3686(17) | 164(3) |

Symmetry transformations used to generate equivalent atoms:
#1 -x+3/2,y+1/2,z    #2 x+1,y,z

## References


(1) Drew, M. G. B.; Hamidbinothman, A.; McIlroy, P. D. A.; Nelson, S. M. 7-Coordination in Metal-Complexes of Quinquedentate Macrocyclic Ligands .2. Synthesis, Properties, and Crystal and Molecular-Structures of Some Iron(III) Derivatives of 2 'N5' Macrocycles. *J. Chem. Soc. Dalton T.* **1975**, 2507-2516.

(2) Drew, M. G. B.; Grimshaw, J.; McIlroy, P. D. A.; Nelson, S. M. 7-Coordination in Metal-Complexes of Quinquedentate Macrocyclic Ligands .3. Preparation and Properties of Some Iron(II) Complexes of 2,13-dimethyl-3,6,9,12,18-penta-azabicyclo-12.3.1 octadeca1(18),2,12,14,16-pentaene and 2,14-dimethyl-3,6,10,13,19-penta-azabicyclo 13.3.1 nonadeca-1(19),13,15,17-pentaene. *J. Chem. Soc. Dalton T.* **1976**, 1388-1394.

(3) Nelson, S. M.; Busch, D. H. 7-Coordination in Some Mononuclear and Binuclear Iron(III) Complexes Containing a Pentadentate Macrocyclic Ring. *Inorg. Chem.* **1969**, *8*, 1859.

(4) Stroka, J. R.; Kandemir, B.; Matson, E. M.; Bren, K. L. Electrocatalytic Multielectron Nitrite Reduction in Water by an Iron Complex. *ACS Catal.* **2020**, *10*, 13968-13972.

(5) *CrysAlisPro*, version 171.41.116a, Rigaku Corporation: Oxford, UK, 2021.

(6) Sheldrick, G. M. SHELXT, Version 2018/2. *Acta Crystallogr.* **2015**, *A71*, 3-8.

(7) Sheldrick, G. M. SHELXL, Version 2018/3. *Acta Crystallogr.* **2015**, *C71*, 3-8.

(8) Dolomanov, O. V.; Bourhis, L. J.; Gildea, R. J.; Howard, J. A. K.; Puschmann, H. *Olex*2, version 1.3-cc4. *J. Appl. Cryst.* **2009**, *42*, 339-341.

(9) Bard, A. J. F., L. R. *Electrochemical Methods: Fundamentals and Applications*; 2nd ed.; John Wiley & Sons, Inc.: New York, 2001.

(10) Savéant, J.-M. *Elements of Molecular and Biomolecular Electrochemistry: An Electrochemical Approach to Electron Transfer Chemistry*; John Wiley & Sons, Inc.: Hoboken, NJ, 2006.

(11) Weatherburn, M. W. Phenol-Hypochlorite Reaction for Determination of Ammonia. *Anal. Chem.* **1967**, *39*, 971-974.

(12) Kolasa, T.; Wardencki, W. Quantitative-Determination of Hydroxylamine. *Talanta* **1974**, *21*, 845-857.

(13) Rapson, T. D.; Church, J. S.; Trueman, H. E.; Dacres, H.; Sutherland, T. D.; Trowell, S. C. Micromolar Biosensing of Nitric Oxide Using Myoglobin Immobilized in a Synthetic Silk Film. *Biosens. Bioelectron.* **2014**, *62*, 214-220.
